# Proteomic Basis of Symbiosis: A Heterologous Partner Fails to Duplicate Homologous Colonization in a Novel Cnidarian– Symbiodiniaceae Mutualism

**DOI:** 10.3389/fmicb.2019.01153

**Published:** 2019-05-31

**Authors:** Emmanuel Medrano, Daniel G. Merselis, Anthony J. Bellantuono, Mauricio Rodriguez-Lanetty

**Affiliations:** Department of Biological Sciences, Florida International University, Miami, FL, United States

**Keywords:** symbiosis, cnidarian, *Exaiptasia pallida*, Symbiodiniaceae, *Symbiodinium linucheae*, *Durusdinium trenchii*, symbiosis core genes

## Abstract

Reef corals and sea anemones form symbioses with unicellular symbiotic dinoflagellates. The molecular circumventions that underlie the successful intracellular colonization of hosts by symbionts are still largely unknown. We conducted proteomic analyses to determine molecular differences of *Exaiptasia pallida* anemones colonized by physiologically different symbiont species, in comparison with symbiont-free (aposymbiotic) anemones. We compared one homologous species, *Symbiodinium linucheae*, that is natively associated with the clonal *Exaiptasia* strain (CC7) to another heterologous species, *Durusdinium trenchii*, a thermally tolerant species that colonizes numerous coral species. This approach allowed the discovery of a core set of host genes that are differentially regulated as a function of symbiosis regardless of symbiont species. The findings revealed that symbiont colonization at higher densities requires circumvention of the host cellular immunological response, enhancement of ammonium regulation, and suppression of phagocytosis after a host cell in colonized. Furthermore, the heterologous symbionts failed to duplicate the same level of homologous colonization within the host, evidenced by substantially lower symbiont densities. This reduced colonization of *D. trenchii* correlated with its inability to circumvent key host systems including autophagy-suppressing modulators, cytoskeletal alteration, and isomerase activity. The larger capability of host molecular circumvention by homologous symbionts could be the result of a longer evolutionary history of host/symbiont interactions, which translates into a more finely tuned symbiosis. These findings are of great importance within the context of the response of reef corals to climate change since it has been suggested that coral may acclimatize to ocean warming by changing their dominant symbiont species.

## Introduction

Coral reefs, frequently referred as “the rainforest of the sea,” represent the most diverse marine ecosystem. Comprising about 0.1% of the earth surface, coral reefs provide habitat for 93,000 known species (Reaka-Kudla, 1997), including more than 800 species of corals (Veron, 2000). Beyond their biodiversity value, coral reefs also provide nearly US $9.9 trillion a year (Costanza et al., 2014) in economic and ecosystem services that directly benefit about 500 million people (Pandolfi et al., 2011). The high biodiversity and productivity of these ecosystems is, at first glance, perplexing considering that coral reef organisms grow in nutrient-poor waters within sub-tropical and tropical latitudes. Corals have thrived in these environments due to highly efficient nutrient cycling provided by their obligate mutualistic relationship with photosynthetic dinoflagellates of the family Symbiodiniaceae (formerly the genus *Symbiodinium* [Muscatine and Porter, 1977; Davy et al., 2012; LaJeunesse et al., 2018]), which live inside the hosting coral’s gastrodermal cells. In this coral-symbiont system, the corals provide their algal symbionts with shelter and nutrients utilized partially for photosynthesis, while the symbionts provide the coral with up to 95% of the fixed carbon they produce (Muscatine et al., 1984).

The coral-dinoflagellate symbiosis is susceptible to disturbance- the loss of the algal symbiont and/or pigmentation, a phenomenon known as coral bleaching, which is provoked when corals experience thermal stress (Hoegh-Guldberg and Smith, 1989; Hoegh-Guldberg, 1999; Venn et al., 2006). With climate change and increasingly warm waters, corals are facing more frequent and severe bleaching events (Hoegh-Guldberg, 1999; Donner et al., 2005; Hoegh-Guldberg et al., 2007). The loss of this obligate symbiosis negatively impacts corals as they suffer from reduced growth rates, impaired reproduction, and tissue necrosis (Harriott, 1985; Goreau and Macfarlane, 1990; Szmant and Gassman, 1990; Glynn, 1993; Baird and Marshall, 2002). In the face of this ecological crisis, several studies conducted over the last 10 years have shown that few coral species have the potential to acclimatize to thermal anomalies by shifting their symbiotic communities from heat-sensitive Symbiodiniaceae species to more thermally tolerant ones (Baker et al., 2004; Berkelmans and van Oppen, 2006). Hosting or changing over to host Symbiodiniaceae of the genus *Durusdinium* (formerly *Symbiodinium* Clade D; LaJeunesse et al., 2018) increases bleaching resistance (Baker et al., 2004; Berkelmans and van Oppen, 2006). Moreover, LaJeunesse et al. (2009) identified a specific thermally tolerant symbiont, *Durusdinium trenchii*. While adaptive bleaching might be considered a nugget of hope for coral reefs under an era of climate change (Berkelmans and van Oppen, 2006), not all corals have the same flexibility for symbiotic engagement with diverse Symbiodiniaceae taxa (Rodriguez-Lanetty et al., 2004, 2006b; Goulet, 2007). However, these thermally tolerant symbionts have been associated with decreased growth rates and opportunism (Little et al., 2004; LaJeunesse et al., 2009; Jones and Berkelmans, 2011), but in at least one coral host, the growth deficit associated with *D. trenchii* colonization disappears at higher temperatures (Cunning et al., 2015). Further, hosting *D. trenchii* may also increase holobiont disease resistance (Rouzé et al., 2016).

The cellular and molecular mechanisms that underlie the successful engagement and maintenance of symbiosis by different Symbiodiniaceae species are still largely unknown (Davy et al., 2012). With the emergence of the sea anemone model system *Exaiptasia pallida* (formerly *Aiptasia pallida*; Grajales and Rodríguez, 2014) to study cnidarian symbiosis, several laboratories have recently characterized the genome and exome of this organism (Sunagawa et al., 2009; Lehnert et al., 2013; Baumgarten et al., 2015) and developed an experimental system to ask fundamental questions of symbiosis (Weis et al., 2008). This anemone possesses not only the capacity to live without Symbiodiniaceae (i.e., aposymbiotic state) but also the flexibility to engage with different Symbiodiniaceae species (Perez et al., 2001). As such, it represents an ideal system to investigate the molecular basis of symbiosis regulation by comparing anemones harboring different Symbiodiniaceae species with anemones lacking the algal symbionts. While previous studies have conducted transcriptomic and proteomic profiling comparisons between symbiotic and aposymbiotic cnidarian hosts (Kuo et al., 2004, 2010; Barneah et al., 2006; Rodriguez-Lanetty et al., 2006a; Voolstra et al., 2009; Ganot et al., 2011; Lehnert et al., 2013; Oakley et al., 2015), these studies have focused the comparison on cnidarians hosting one species of Symbiodiniaceae, with the exception of one recent study (Matthews et al., 2017).

The present study leverages the power of comparing different symbiont identities as well as presence and absence of symbiont (symbiotic state) in a clonal host background. Here, we use a proteomic approach to determine differences within the protein profiles of these anemones hosting physiologically distinct Symbiodiniaceae species in comparison with aposymbiotic anemones. We chose to compare one homologous species, *Symbiodinium linucheae*, that is natively endogenous to the clonal *E pallida* strain (CC7) to another heterologous species, *Durusdinium trenchii*, a thermally tolerant species that colonizes numerous coral species (Bieri et al., 2016). Furthermore, we expanded upon the contrast between symbiont identity and symbiotic state by exploring diurnal proteomic dynamics. Our results demonstrate the presence of a core set of host genes that are differentially regulated as a function of symbiosis regardless of the species of Symbiodiniaceae associated with the host. This study also identifies proteomic changes specific to symbiont identity which may underlie the mechanistic basis of the failure of the heterologous symbionts to colonize the host to the same extent as a homologous species.

## Results

### Symbiodiniaceae Colonization in *Exaiptasia pallida* After a Year of Symbiotic Engagement

**FIGURE 1:**
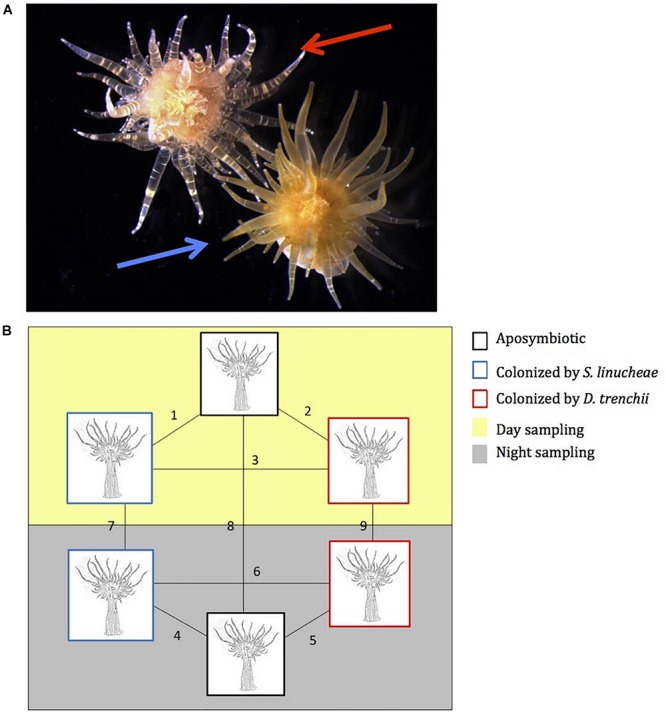
Experimental host anemones, *Exaiptasia pallida*. **(A)** Photographs of 1-year-old anemones colonized by *Symbiodinium linucheae* (blue arrow) and by *Durusdinium trenchii* (red arrow). **(B)** Pairwise experimental design for proteomic comparison analysis between symbiotic and aposymbiotic anemones. Black box: aposymbiotic, blue box: colonized by *S. linucheae*, and red box: colonized by *D. trenchii*. Yellow panel: sampled during the day (12 p.m.); and gray panel: sampled at night (12 a.m.).

**FIGURE 2:**
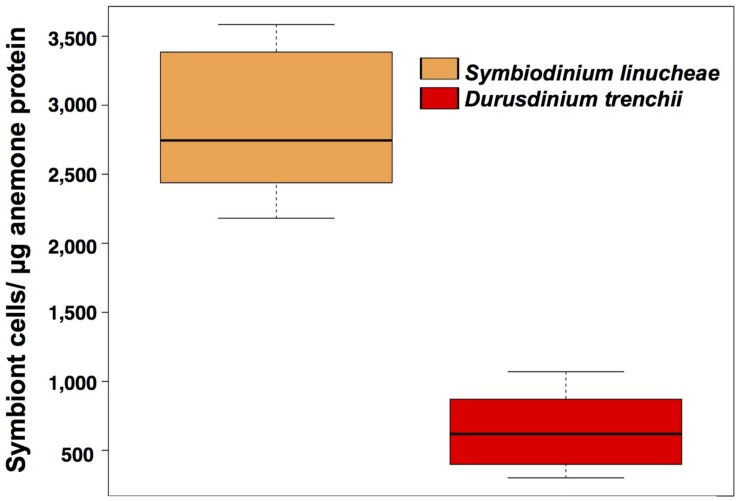
Symbiodiniaceae density normalized by total holobiont protein from anemones colonized by different symbionts. These measurements were taken after 1 year of successful symbiont colonization (*N* = 20). Orange: *Symbiodinium linucheae*; red: *Durusdinium trenchii.*

One year after the onset of symbiosis (Figure 1A), the two different Symbiodiniaceae species maintained significantly different cell densities within the experimental CC7 host anemones (Figure 2). The colonization density by the homologous symbionts, *Symbiodinium linucheae* (2.87 ± 0.60 × 10^3^ cells μg^–1^ protein) was four times denser than that of the heterologous symbiont, *Durusdinium trenchii* (0.65 ± 0.32 × 10^3^ cells μg^–1^ protein). The level of endosymbiotic dinoflagellate densities within *Exaiptasia pallida* anemones reported in this study and the colonization differences between the two species of Symbiodiniaceae are consistent with values documented by other studies for homologous vs. heterologous symbionts (Leal et al., 2015; Sproles, 2017).

### Magnitude of Host Proteomic Alterations as a Function of Symbiodiniaceae Species

The proteomic profiles of symbiotic anemones colonized by different Symbiodiniaceae species (*S. linucheae* and *D. trenchii*) were compared in reference to proteomes of aposymbiotic anemones (those lacking algal symbionts). The comparisons were conducted in anemones sampled both during day (solar noon) and during night (solar midnight). From these four comparisons (two symbionts species × two sampling times), 97 differentially expressed unique host proteins were identified among approximately 1,600 protein features visibly detected on the 2D DIGE gels. The data reveal a core proteome of symbiosis; that is, both homologous *S. linucheae*- and heterologous *D. trenchii*-hosting anemones shared expression commonalities in comparison with aposymbiotic anemones, but also exhibited distinct symbiont-specific responses both during the day and night, as described below.

### *Exaiptasia pallida* Displays Upregulation of Core Proteins as a Function of Symbiosis

**FIGURE 3:**
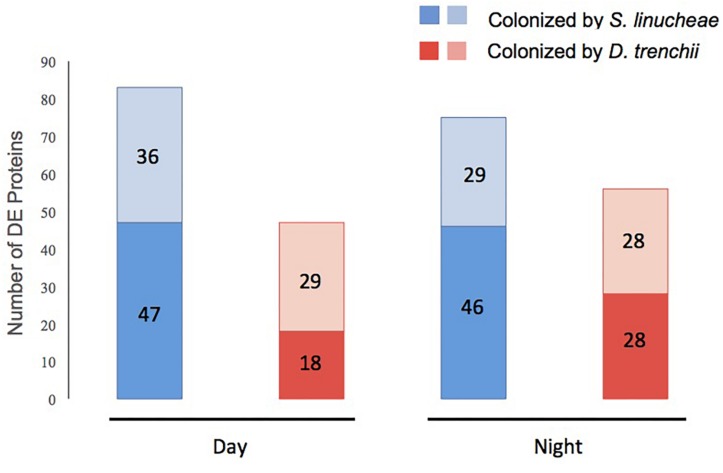
Total number of differentially expressed (DE) proteins within each group of symbiotic *Exaiptasia pallida* anemones colonized by homologous and heterologous Symbiodiniaceae symbionts compared to aposymbiotic anemones. Proteins up-regulated are in dark color and those down-regulated in light color. Blue bars show proteins in anemones colonized by the homologous *S. linucheae* symbionts; while red bars represent proteins in anemones colonized by the heterologous *D. trenchii*. The comparisons were carried out both in the day and at night.

**FIGURE 4:**
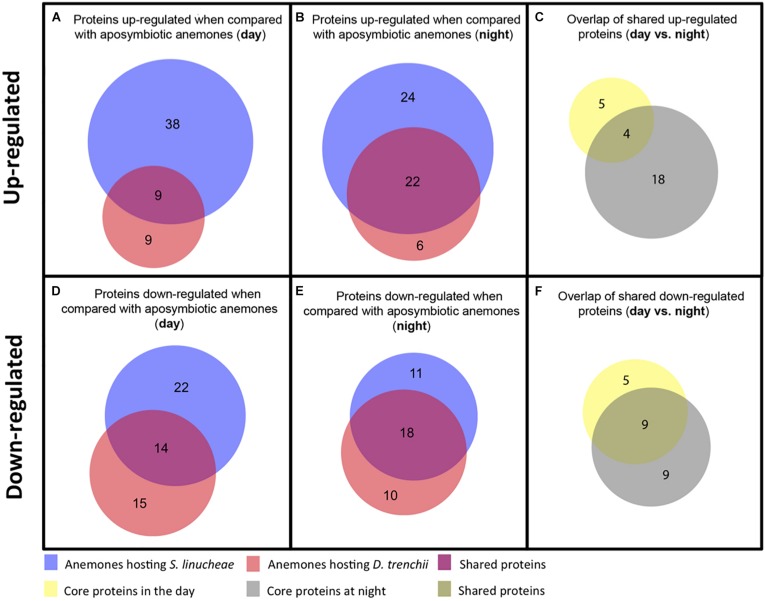
Venn diagrams showing the number of up-regulated and down-regulated proteins that overlap between anemones colonized by different species of Symbiodiniaceae. **(A)** Overlap of up-regulated proteins from anemones colonized by *S. linucheae* or *D. trenchii* sampled in the day. **(B)** Overlap of up-regulated proteins from anemones hosting the two species of symbionts sampled at night. **(C)** Overlap of shared proteins from day **(A)** vs. night **(B)**. **(D)** Overlap of down-regulated proteins sampled in the day. **(E)** Overlap of down-regulated proteins sampled at night. **(F)** Overlap of shared proteins from day **(D)** vs. night **(E)**.

During the day, anemones colonized by homologous *S. linucheae* differentially up-regulated more than twice as many proteins in reference to the aposymbiotic group than those colonized by heterologous *D. trenchii* (*n* = 47 and *n* = 18, respectively; Figure 3). A further comparison of these two datasets revealed nine “core symbiosis” proteins that were shared between these symbiotic anemone groups and are necessary for symbiosis regardless of symbiont identity (Figure 4A and Supplementary Table S1). Similarly, at night the anemones colonized by the homologous symbiont up-regulated twice as many host genes as the anemones colonized by the heterologous symbiont relative to the aposymbiotic group (Figure 3). At night there was a higher proportion of core symbiosis proteins (*n* = 22/52) shared between symbiotic anemones compared to the day-sampled anemones (*n* = 9/56) (Figures 4A,B). The overlap found for anemones sampled at night also suggests the presence of a core set of proteins.

Core symbiotic proteins from the day (*n* = 9) and night (*n* = 22) groups shared an overlap of four proteins (Figure 4C and Table 1). These four represent the core up-regulated proteins that are found in symbiotic anemones regardless of symbiont type or day/night sampling group. The remaining proteins correspond to either day- or night-specific up-regulated proteins (Table 1).

**TABLE 1:** List of proteins up-regulated regardless of symbiont species identity under day and night.

**Proteins up-regulated in symbiotic *Exaiptasia pallida* anemones (day)**	
**Gel ID**	**Protein**
20	Failed axon connections-like
23	Failed axon connections-like
68	Actin, cytoplasmic
91	Actin, cytoplasmic 1
129	Protein NPC2-like

**Proteins up-regulated in symbiotic anemones (night)**	
**Gel ID**	**Protein**

2	Myosin heavy chain, striated muscle
3	Endoplasmin
11	Calpain-B
18	Protein disulfide-isomerase
24	ATP synthase subunit beta, mitochondrial
33	Phenylalanine–tRNA ligase alpha subunit
39	Glutamate dehydrogenase
46	Actin, cytoplasmic
47	Actin, cytoplasmic
48	Major antigen
70	Phosphoethanolamine *N*-methyltransferase 3
75	Translationally controlled tumor protein-like
76	14-3-3-like protein 2
77	14-3-3 protein zeta
80	Rho GDP-dissociation inhibitor 1
90	GTPase KRas
94	Major antigen
140	E3 ubiquitin-protein ligase DZIP3

**Proteins up-regulated in symbiotic anemones regardless of day/ night sampling**	
**Gel ID**	**Protein**

40	Glutamate dehydrogenase
42	Gelsolin-like protein 2
43	Gelsolin-like protein 1
106	Myosin regulatory light chain 12B

*Proteins with known functional annotations are reported.*

### *Exaiptasia pallida* Also Displays Downregulation of Core Proteins as a Function of Symbiosis

The proteomic profile of anemones sampled during the day also showed downregulation of many proteins as a function of symbiosis (Figure 3). Anemones colonized by homologous *S. linucheae* down-regulated 36 proteins in reference to aposymbiotic anemones, which represented 43% of the total that were differentially expressed within the homologous anemone treatment (Figure 3). In contrast, anemones colonized by heterologous *D. trenchii* showed a proportionally greater (62%) downregulation (Figure 3). These results demonstrate an overrepresentation of down-regulated proteins compared to those up-regulated in anemones colonized by the heterologous endosymbiont, which is opposite the pattern in anemones colonized by the homologous symbionts. Anemones colonized either by *S. linucheae* or *D. trenchii* showed a common set of 14 proteins (Figure 4D) that represent a core set of down-regulated proteins in *E. pallida* as a result of symbiosis during the day.

A similar proteomic profile was present in anemones sampled at night. Anemones colonized by homologous *S. linucheae* down-regulated 29 proteins while anemones colonized by heterologous *D. trenchii* showed decreased expression in 28 proteins (Figure 3). Although the absolute number of differentially down-regulated proteins was similar, anemones colonized by *D. trenchii* again exhibited a proportionally greater number of down-regulated proteins than those colonized by the homologous symbionts, 50% (28/56) and 39% (29/75), respectively (Figure 3). Both groups of anemones shared a set of 18 core proteins in reference to aposymbiotic anemones at night, a larger proportional overlap of differentially expressed proteins during night than during daytime, 46% and 27%, respectively (Figure 4E). A total of nine proteins were shared between the core set that were down-regulated in the day (*n* = 14) and at night (*n* = 18) indicating that these proteins represent a core set that are regulated independently of symbiont type and day/night group (Figure 4F and Table 2).

**TABLE 2:** List of proteins down-regulated regardless of symbiont species identity under day and night.

**Proteins down-regulated in symbiotic *Exaiptasia pallida* anemones (day)**	
**Gel ID**	**Protein**
1	Myosin heavy chain, striated muscle
45	Heat shock cognate 71 kDa protein
69	Inactive pancreatic lipase-related protein 1
84	*S*-crystallin 4
121	Ectin

**Proteins down-regulated in symbiotic anemones (night)**	
**Gel ID**	**Protein**

21	Lysostaphin
26	Tubulin alpha-1C chain
31	Selenium-binding protein 1
36	Hypothetical protein AC249_AIPGENE13469
50	Actin, cytoplasmic
56	Serine–pyruvate aminotransferase, mitochondrial
59	Malate dehydrogenase, cytoplasmic
82	Actin, cytoplasmic
128	Rho GDP-dissociation inhibitor 1

**Proteins down-regulated in symbiotic anemones regardless day/ night sampling**	
**Gel ID**	**Protein**

8	Hypothetical protein AC249_AIPGENE16477
13	Phosphoenolpyruvate carboxykinase (GTP), mitochondrial
19	Neuronal pentraxin-2
37	Calpain-B
38	Alpha-enolase
44	Fumarylacetoacetase
79	14-3-3 protein zeta
112	ADP-ribosylation factor 1
122	Hypothetical protein AC249_AIPGENE3884

*Proteins with known functional annotations are reported.*

### Differences in Proteomic Profiles of Anemones Colonized by Homologous *S. linucheae* Versus Those Colonized by Heterologous *D. trenchii*

Differentially expressed proteins that did not belong to a core symbiosis set fall within those that were specific to the type of Symbiodiniaceae that colonized the anemone (Table 3). Within this case, we identified 11 proteins up-regulated in anemones colonized by the homologous *S. linucheae* but these were down-regulated in anemones containing the heterologous *D. trenchii*. Conversely, there were four proteins up-regulated in hosts colonized by *D. trenchii* but down-regulated in those colonized by *S. linucheae*.

**TABLE 3:** List of proteins up-regulated (↑) in *Exaiptasia pallida* anemones colonized by homologous *Symbiodinium linucheae* but down-regulated (↓) in anemones colonized by heterologous *Durusdinium trenchii*.

**Gel ID**	**Sampling time**	**Sampling time**	**Protein**
	***S. linucheae***	***D. trenchii***	
2	Day ↑	Day ↓	Myosin heavy chain, striated muscle
3	Day ↑	Day ↓	Endoplasmin
18	Day ↑	Day ↓	Protein disulfide-isomerase
94	Day ↑	Day ↓	Major antigen
119	Day ↑	Day/night ↓	Hypothetical protein
92	Night ↑	Day/night ↓	Golgi-associated plant pathogenesis-related protein 1
93	Night ↑	Day/night ↓	Sorcin
16	Night ↑	Night ↓	Hypothetical protein
4	Day/night ↑	Day ↓	Spectrin
78	Day/night ↑	Day ↓	Peptidyl-prolyl *cis–trans* isomerase
132	Day/night ↑	Day ↓	Hypothetical protein

*The pattern of expression is compared to symbiont-free anemones (aposymbiotic state). It is also indicated the sampling time of the day when the difference was detected for anemones colonized by either symbiont species.*

### Proteomic Differences Between Day and Night

**FIGURE 5:**
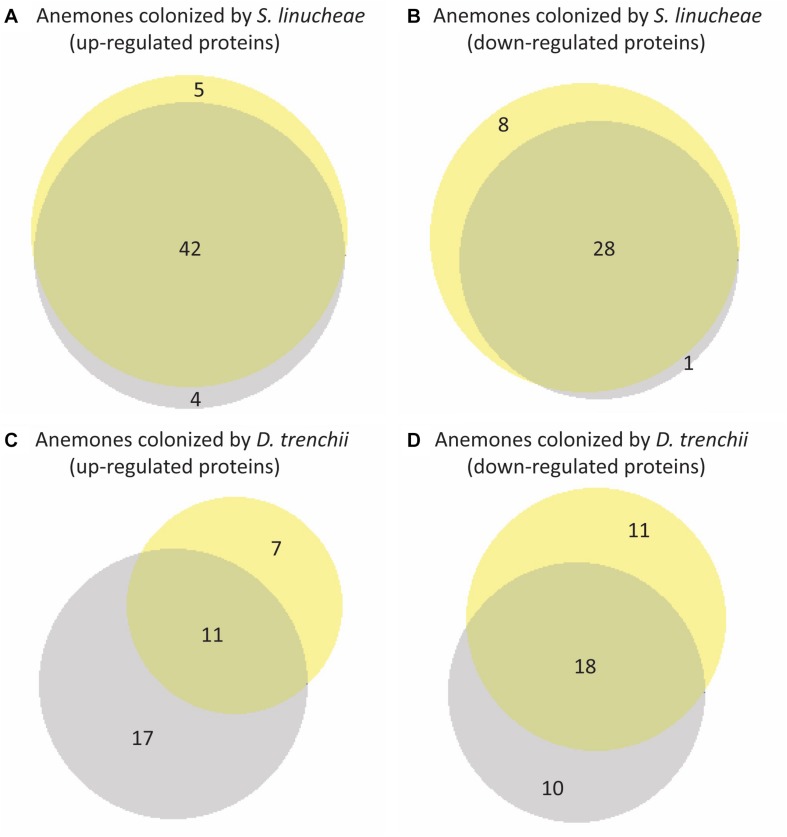
Venn diagrams showing overlap of differentially expressed proteins within each group of anemones between day and night. Yellow represents proteins sampled in the day and gray represents night samples. **(A)** Overlap of proteins up-regulated from day and night conditions of anemones colonized by *Symbiodinium linucheae.* **(B)** Overlap of proteins down-regulated from day and night conditions of anemones colonized *S. linucheae.* **(C)** Overlap of up-regulated proteins from day and night conditions of anemones colonized by *Durusdinium trenchii.* **(D)** Overlap of proteins down-regulated from day and night conditions of anemones colonized by *D. trenchii.* The matching list of host proteins displayed in this figure is provided in Supplementary Table S2.

The expression profile of anemones colonized by the homologous *S. linucheae* in reference to aposymbiotic anemones remained largely constant through the entire day with regard to up-regulated genes, with 82% (42/51) of up-regulated proteins concordant between day and night (Figure 5A). Similar results were observed for the corresponding comparison of down-regulated proteins, with 76% (28/37) of differentially expressed proteins in concordance (Figure 5B). In contrast, the profile of anemones colonized by the heterologous *D. trenchii* was highly dynamic between day and night, with just 31% (11/35) concordance between up-regulated proteins and 46% (18/39) in down-regulated (Figures 5C,D).

## Discussion

Notable differences in Symbiodiniaceae cell density in anemones colonized by homologous and heterologous species of endosymbionts, grown under identical laboratory conditions, indicate that the process of symbiont colonization was not equal. The homologous symbiont *S. linucheae* was able to achieve higher colonization density after a year of symbiotic engagement than the heterologous symbiont *D. trenchii*. These results are consistent with a previous study conducted on a geographically disparate clone of *E. pallida* anemones (NZ1) from New Zealand where the heterologous symbiont *Durusdinium trenchii* colonized in considerably lower densities than the homologous *Breviolum minutum* (Sproles, 2017). Similarly, colonization comparison between heterologous symbionts with the CC7 anemone strain as host also showed that *D. trenchii* attained the lowest densities in comparison with numerous strains of heterologous *B. minutum* (Leal et al., 2015). Only one study showed comparable levels of colonization between heterologous *D. trenchii* and homologous *B. minutum* using the NZ1 anemone strain (Matthews et al., 2017). However, as opposed to a long-term, established symbiosis achieved over the course of months or years, the experimental anemones from this recent study by Matthews et al. (2017) were sampled 1 week after 4 consecutive weeks of symbiont re-inoculations. Under this inoculation approach it is likely that the dinoflagellates associated with the anemone host represented a mixed population of symbiotic stages including recently engulfed symbionts along with some dinoflagellates not engaged in stable endosymbiosis.

By clearly identifying a different level of symbiont colonization after a year of steady symbiotic interactions between homologous and heterologous symbionts, we were able to interrogate the molecular alterations in these different holobiont assemblages through comparison with aposymbiotic anemones (i.e., lacking of endosymbiotic dinoflagellates). An overarching pattern of differential protein expression detected in these Symbiodiniaceae-specific comparisons was a dramatically larger number of proteins regulated as a function of symbiosis (i.e., in reference to aposymbiotic anemones) in anemones colonized by the homologous symbiont compared to those colonized by the heterologous symbiont. This finding supports similar patterns discovered in a different population of *Exaiptasia pallida* anemones from the Pacific (natively hosting *B. minutum*) relative to the same heterologous species (*D. trenchii*) (Matthews et al., 2017). The lower level of cellular alterations in the anemones colonized by the heterologous *D. trenchii* may correlate with life history traits of this symbiont such as being an opportunistic colonizer after bleaching events due to its thermal tolerance characteristics (Little et al., 2004; Baker et al., 2008; Silverstein et al., 2017) that provides fewer nutritional benefits than the native symbiont species (Pettay et al., 2015).

We also discovered a congruent pattern of the symbiosis-regulated proteins between day and night in those anemones colonized by the homologous symbionts. By contrast, the anemones colonized by the heterologous symbionts share fewer differentially expressed proteins between day and night, which indicates a more dynamic pattern of expression. This dynamism of expression shows the existence of a diel regulation pattern in the anemones colonized by the heterologous symbiont. While the cause of this remains to be resolved, it is possible the heterologous partners more drastically alter expression levels to maintain homeostasis relative to homologous symbionts that drive a more constant state of host gene regulation.

Despite these differences, a minimum of essential molecular circumventions is expected to occur for any symbiotic dinoflagellates to take residence inside a cnidarian the host cell. We were able to identify common core modulations of protein expression in response to symbiosis when colonized with either homologous or heterologous symbionts. Protein expression comparison between day and night provided further compelling evidence of the role of these core proteins in symbiosis. Below we discuss these shared molecular alterations between anemones colonized by homologous and heterologous symbionts. We also argue that lack of some alterations in those anemones colonized by the heterologous symbionts might be linked to differences in symbiotic engagement.

### Endosymbiosis Requires Circumvention of Cellular Immunological Response

Our results show high expression of two gelsolin-like proteins during day and night in symbiotic anemones regardless of the type of colonized symbionts (Table 1 and Supplementary Table S1). This discovery is in congruence with recent findings also showing an upregulation of a gelsolin-like protein in symbiotic anemones that were sampled during the day (Sproles, 2017). Gelsolin proteins are calcium-regulated members of a large superfamily of actin binding proteins (McGough et al., 2003) that serve as key regulators of actin filament assembly and disassembly (Sun et al., 1999). Apart from its role in cytoskeleton arrangement, gelsolin has also been shown to inhibit apoptosis by blocking voltage-dependent anion channel (VDAC) activity in mammals, thus preventing the release of cytochrome *c* from the mitochondria and activation of caspases 3, 8, and 9, signaling that otherwise would lead to apoptosis (Koya et al., 2000; Kusano et al., 2000). If the same cellular function of gelsolin documented in mammals also exists in cnidaria, it would be possible that gelsolin may play a crucial role in suppressing apoptosis in symbiotic cnidarian host cells. This finding would support our previously postulated model that suppression of apoptosis, together with deregulation of the host cell cycle, creates a platform that might be necessary for symbiont and/or symbiont-containing host cell survival (Rodriguez-Lanetty et al., 2006a). In support of this hypothesis, gelsolin transcripts were found to be down-regulated in thermally stressed corals to bleaching (Desalvo et al., 2010), which strengthens the postulation of the importance of gelsolin in symbiotic stability. The discovery of gelsolin-like proteins as constitutively expressed core symbiosis modulators adds a new piece to the puzzle of the on-going developing molecular model that explains the regulation of cnidarian/Symbiodiniaceae symbiosis.

### Ammonium Regulation Is Enhanced During Symbiosis and May Involve Host Upregulation of a Key Gene in Exchange of Algal Translocates

Another protein significantly up-regulated during day and night in symbiotic anemones independent of symbiont identity was glutamate dehydrogenase (Table 1 and Supplementary Table S1). This enzyme drives the reversible conversion between glutamate and α-ketoglutarate; in animals the enzyme favors the production of α-ketoglutarate and the secondary product, ammonia (Spanaki and Plaitakis, 2012). The capacity of both symbiont and host to assimilate ammonium has been firmly established (Wang and Douglas, 1998; Yellowlees et al., 2008), and the involvement of glutamate dehydrogenase, an enzyme implicated ammonium regulation, would be expected as a core symbiosis gene. Early work by Muscatine and D’Elia (1978) reported enhanced ammonium assimilation rates in the intact symbioses under light, and more recent studies demonstrate that dinoflagellate symbionts can fix between 10 and 23 times more nitrogen than coral host cells in response to a sudden pulse of ammonium-enriched seawater (Grover et al., 2002; Pernice et al., 2012). In nutrient-poor (oligotrophic) environments, the typical setting where tropical coral reefs flourish, the photosynthetic symbiotic dinoflagellate is nitrogen-limited (Muscatine and Porter, 1977; Rädecker et al., 2015), which has been the basis for the argument that the animal host controls endosymbiont populations by regulating nitrogen supply (Falkowski et al., 1993). Since α-ketoglutarate, produced from glycolysis-derived products that enter the TCA (tricarboxylic acid) cycle, gets metabolized and consumed as the TCA cycle proceeds, glutamate dehydrogenase will favor the chemical conversion of glutamate into α-ketoglutarate with ammonium as a byproduct. Consequently, the ammonium will be rapidly consumed by the algal symbionts and the host will take advantage of the extra α-ketoglutarate to further fuel the TCA cycle. We posit that two major nutritional arrangements take place between the host and endosymbiont. Under conditions of stable symbiosis, the host will maintain high expression of glutamate dehydrogenase and produce ammonium so long as *in hospite* dinoflagellates sustain the translocation of carbohydrates such as glucose to the host, which feeds the host TCA cycle after glycolysis.

### Phagocytosis May Be Reduced Following Symbiotic Engagement

We identified a downregulation in ADP ribosylation factor 1 (ARF1) during day and night (Table 2 and Supplementary Table S1), consistent with a previous Symbiodiniaceae/cnidarian association study (Chen et al., 2004). ARF1 has been implicated in intracellular trafficking during receptor-mediated endocytosis, endosomal recycling, and exocytosis of secretory granules (Amor et al., 1994). Its inhibition in a murine cell line of macrophages results in the early blockade of pseudopod extension and accumulation of intracellular vesicles, followed by a reduction of phagocytosis (Niedergang et al., 2003). While no functional implication has been suggested for ARF1 in Symbiodiniaceae/cnidarian symbioses, we proposed that the down-regulation of ARF1 during symbiosis may be involved in suppressing phagocytosis following the symbiotic engagement of a host cell. This model agrees with cellular observations during the establishment of symbiosis between a jellyfish and Symbiodiniaceae (Colley and Trench, 1985). Colley and Trench (1985) discovered that within 3 days after initial dinoflagellate uptake, most endodermal cells with algae ceased phagocytosis. By decreasing or abolishing phagocytosis in a host cell, a recently colonizing symbiont ensures the host cell does not engulf another algal symbiont that would compete for the resources and available space inside the hosting cell. While reduction of phagocytosis might be of concern for the host from a nutritional perspective (Davy et al., 2012), it is likely offset by photosynthate translocation. This modulation of phagocytosis as a function of symbiosis seems to occur regardless of the Symbiodiniaceae species that colonize the cnidarian host cell.

### Other Important Genes Required in Symbiosis With Dynamic Diel Expression

Unlike the constitutively differentially regulated genes discussed above, dynamically regulated genes respond to physiological changes pertinent to each symbiont player occurring during the diel cycle. Sterol-trafficking Niemann–Pick type C proteins, NPC1 and NPC2, are examples of this phenomenon, having recently been implicated in cnidarian-dinoflagellate symbiosis (Kuo et al., 2010; Ganot et al., 2011 and also reviewed in Davy et al., 2012). In mammalian and *Drosophila* cells, NPC2 binds cholesterol in the lumen of the endosome and lysosome and transfers it to NPC1, a transmembrane protein that exports the cholesterol to other intracellular locations. The *Anemonia viridis* NPC2 (AvNPC2-d) was found to be up-regulated in symbiotic relative to aposymbiotic sea anemones and its expression was indeed higher in the gastrodermis (symbiont-containing tissue) closely localized with symbiosomes (Ganot et al., 2011; Dani et al., 2014, 2017). These findings of up-regulation of NPC2 transcript in the symbiotic state has been confirmed in other cnidarian hosts such as *Aiptasia pulchella* (Voolstra et al., 2009) and *Exaiptasia pallida* (Lehnert et al., 2013; Oakley et al., 2015; Sproles, 2017). Collectively, these findings suggest that NPC2-d might be involved in traffic of various dinoflagellate-produced sterols but also in the stability and dysfunction of cnidarian-dinoflagellate symbioses as its expression decreases under hyperthermal conditions (Ganot et al., 2011). Our data bolstered these findings by demonstrating that regardless of the symbiont type harbored, the host upregulates the expression of NPC2 in the symbiotic state (Table 1 and Supplementary Table S1). We also discovered that the expression was higher during the day (6 h after sunrise) but not differentially expressed at night (6 h after sunset) (Supplementary Table S1). This daily cycle of NPC2 expression may be a response to varying levels of photosynthate as a consequence of diel patterns in photosynthetic activity.

**FIGURE 6:**
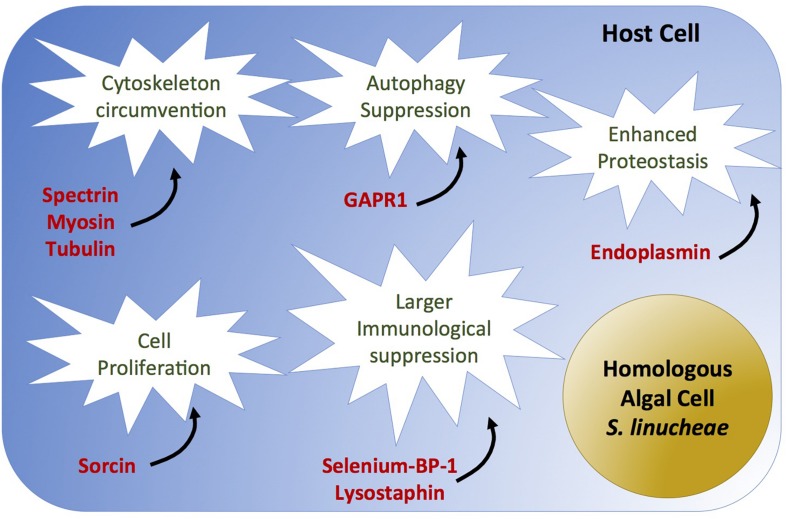
Causal diagram of hypothetical scenario of homologous Symbiodiniaceae-specific circumvented biological process (white stars) and overexpressed proteins (red) in anemones colonized by the homologous *Symbiodinium linucheae*. Anemones colonized by the heterologous *D. trenchii* did not show differential expression of these proteins as a function of symbiosis.

In addition to the aforementioned essential molecular circumventions that appear necessary for any Symbiodiniaceae type to take residence inside a cnidarian host cell, we also discovered that the heterologous *D. trenchii* fails to circumvent other key host proteins that might be needed for more extensive symbiosis colonization. Despite stable engagement, host and heterologous symbiont interactions appear to be poorly tuned as evidenced by the substantial reduction in symbiont density. This differential success in colonization may be explained by a varying ability to modulate specific host genes including those involved in escape from host immune response. Our study identifies proteins that were up-regulated in anemones colonized by homologous symbionts, but down-regulated (or unchanged) in the anemones colonized by the heterologous symbionts. These data suggest that *D. trenchii* failed to circumvent key host systems including autophagy-suppressing modulators, cytoskeletal alteration, and isomerase activity (Figure 6 and Table 3).

### Heterologous Symbionts Fail to Suppress Autophagy

Though cell death circumvention is needed regardless of symbiont identity, the homologous symbiont appears to achieve higher immune tolerance in the host cell by circumventing autophagy in addition to apoptosis. Autophagy serves to protect organisms against diverse pathologies, including microbial infections, cancer, neurodegeneration, aging, and heart disease (Levine and Kroemer, 2008; Mizushima et al., 2008). We discovered that the negative autophagy regulator GAPR-1 (Golgi-associated plant pathogenesis related protein; Shoji-Kawata et al., 2013; Li et al., 2017) was highly expressed in hosts colonized by homologous symbionts, whereas it was significantly down-regulated in *D. trenchii*-colonized anemones (Table 3, Supplementary Table S1, and Figure 6). This suggests a potential higher autophagy capacity/activity in the anemones hosting the heterologous symbionts and therefore less immune tolerance. We postulate that high density colonization by Symbiodiniaceae requires suppression of autophagy, potentially creating a cell survival state analogous to immortalization of cancer cells (reviewed by Levine and Kroemer, 2008; Li et al., 2017). These results align with recent evidence that also show higher immunotolerance related to other immune-related pathways in hosts containing homologous symbionts (Matthews et al., 2017).

### Host Cytoskeleton Modification Differs as a Function of Symbiodiniaceae Species

Signatures of cytoskeleton modification were strongly detected in symbiotic anemones regardless of the type of colonizing symbiont (as shown in Table 1), but distinctions were observed in the type of cytoskeleton proteins involved between anemones colonized by the homologous and heterologous symbionts. These distinctions could relate to the differential success of symbiont colonization of the host’s gastrodermal tissue. For instance, spectrins are key structural proteins that lie just beneath the plasma membrane of eukaryotic cells, providing structural support and protein-sorting capabilities to the membrane (Baines et al., 2009). The spectrin cytoskeleton is crucial for the invasion of the intracellular bacterial pathogens *Salmonella typhimurium* and *Listeria monocytogenes* of mammalian intestinal epithelial cells (Ruetz et al., 2011). The pathogen *Shigella flexneri* utilizes the spectrin cytoskeleton during invasion and dissemination throughout host intestinal epithelial tissues (Ruetz et al., 2012). Our results suggest that the spectrin cytoskeletal network may require modulation by the colonizing Symbiodiniaceae to effectively occupy and spread through the host gastrodermal tissue. Spectrin was up-regulated in anemones colonized by the homologous symbionts in this study and by Oakley et al. (2015, 2017), but down-regulated in the anemones hosting the heterologous symbionts (Table 3, Supplementary Table S1, and Figure 6). Together, these results suggest a role of spectrin in the increased colonization process of homologous Symbiodiniaceae through the host tissue and indicate that *D. trenchii* is not be able to manipulate this cytoskeletal network, which might account for its lower cell density in anemone hosts.

### Isomerase Proteins May Be Important for Symbiosis and Heterologous Symbionts Fail to Modulate Their Expression

We identified an isomerase protein, peptidyl-prolyl *cis–trans* isomerase (PPIase), which was up-regulated during both day and night in *S. linucheae*-hosting anemones, but down-regulated in anemones hosting the heterologous symbiont *D. trenchii* during the day (Table 3, Supplementary Table S1, and Figure 6). PPIases are cyclophilins, acting as molecular switches in multiple cellular processes, such as cellular signal transduction, adaptation to stress, control of pathogen virulence, and modulation of host immune response (Dimou et al., 2017). While there is a well-documented involvement of PPIases in host/pathogen interactions (Coaker et al., 2006; Perez, 2007; Krücken et al., 2009; Yau et al., 2010), there are only a few indications of the connection of PPIases in symbioses regulation, including up-regulation in symbiotic vs. aposymbiotic *Exaiptasia* (Rodriguez-Lanetty et al., 2006a). Inhibition of PPIases in symbiotic *Exaiptasia pallida* anemones with the use of cyclosporin A showed a dose-dependent disruption of the symbiosis with Symbiodiniaceae, resulting in the release of the symbiotic algae from host tissues (Perez, 2007). Our findings further suggest the involvement of PPIases in host/symbiont regulation. Higher expression of peptidyl-propyl isomerase was detected in anemones colonized by homologous symbionts, which might stabilize the symbiotic relationship by isomerizing important phosphorylated proteins involved in symbiosis. By contrast, the lack or low expression of these isomerases in heterologously colonized anemones could be linked to low symbiont density.

## Conclusion

The powerful comparative approach used in this study allowed us to discover the existence of a core set of host genes that are differentially regulated as a function of symbiosis regardless of the colonized species of symbionts. More specifically, the data revealed two major findings. First, the success of both homologous and heterologous symbionts in the colonization of a host requires circumvention of the host cellular immunological response, enhancement of ammonium regulation, and suppression of phagocytosis following symbiotic engagement within a host cell. Second, hosts colonized by homologous symbionts showed more extensive molecular alterations, correlating with greater symbiont colonization relative to the heterologous symbiont. It appears that the relatively low density of the heterologous symbiont, *D. trenchii*, may be explained by its inability to further circumvent processes related to autophagy-suppressing modulators, cytoskeletal alteration, and isomerase activity. We argue that the larger capability of host molecular circumvention by homologous symbionts could be the result of a longer evolutionary history of host/symbiont interactions, producing a more finely tuned symbiosis.

## Materials And Methods

Clonal groups (CC7) of *Exaiptasia pallida* anemones harboring one of two distinct species of Symbiodiniaceae were used in this study to assess differences and commonalities in proteomic profiles of host anemones as a function of symbiont identity and state (symbiotic or aposymbiotic). These anemones host either the homologous (native) *Symbiodinium linucheae* (Bieri et al., 2016), which are frequently associated with *E. pallida* anemones from Florida, or the heterologous (not associated with *Exaiptasia* in nature) *Durusdinium trenchii*, well-known for its ability to convey thermal resistant attributes to the coral holobiont (LaJeunesse et al., 2009; Silverstein et al., 2014). An aposymbiotic anemone population (lacking algal symbiont) was also utilized as a reference for proteomic comparisons.

### Culture Conditions of *Exaiptasia pallida* Anemones

Stock anemones were kept at 26°C room in glass bowls containing autoclaved artificial seawater (ASW) (Coral Pro Salt; Red Sea Aquatics Ltd., Houston, TX, United States) at 32–34 PSU. Anemones were grown under white fluorescent light (intensity of ∼80 μmol photons m^–2^s^–1^) on a diurnal 12 h light:12 h dark cycle, and fed twice weekly with freshly hatched *Artemia franciscana* nauplii. Seawater was changed 6–12 h after feeding.

### Establishment of Symbiotic Anemone Treatment Groups

Aposymbiotic *E. pallida* were created by repetitive cold treatments to remove their native symbionts (*Symbiodinium linucheae*), with remnant symbionts removed via DCMU treatment. Following 1 week of feeding cessation, anemones were incubated in darkness at 4°C for 8 h and then returned to room temperature. Daily water changes with autoclaved ASW were performed during this process. The cold treatment was repeated three times over the course of a week, with 24–48 h between treatments to allow for host recovery. Following cold treatments, anemones were incubated in 50 μM of the herbicide and PSII inhibitor 3-(3,4-dichlorophenyl)1-1 dimethylurea (DCMU) in autoclaved ASW and exposed to 1,000 μmol m^–2^s^–1^ of light for 6 h. After DCMU exposure, ASW containing DCMU was exchanged for autoclaved ASW without DCMU. The DCMU and light exposure treatments were repeated twice weekly for 3 weeks. Loss of symbionts was confirmed using fluorescence microscopy and the absence of detectable PCR amplification of Symbiodiniaceae chloroplast 23S DNA compared with an amplified positive control (Santos et al., 2002; Lewis et al., 2019). Further, aposymbiotic anemones did not spontaneously recolonize with symbionts even after 12 months of maintenance under 12:12 light cycles with no additional DCMU or cold treatments.

A portion of the aposymbiotic anemones were colonized with the non-native, heterologous *Durusdinium trenchii* (Strain CCMP2556) obtained from the National Center for Marine Algae and Microbiota (NCMA) and grown in silica-free Prov50 medium. Log phase *D. trenchii* were mixed with *A. franciscana* nauplii and fed to the anemones to achieve colonization. Colonization was confirmed by fluorescence microscopy, chloroplast 23S DNA PCR amplification, and Sanger sequencing. Stocks of aposymbiotic anemones and anemones infected with *D. trenchii* were increased through asexual reproduction via pedal laceration (Clayton, 1985). Aposymbiotic, heterologous-hosting (*Durusdinium trenchii*), and homologous-hosting (*Symbiodinium linucheae*) anemones were maintained under similar culture conditions for the next 12 months; that is, without any additional DCMU/stress treatments or re-inoculation with symbionts, to ensure that the symbiotic states or lack thereof were stable. While DMCU could have an effect on downstream proteomic analyses, it is important to note that an entire year elapsed between the bleaching of anemones with photosynthetic inhibitor DMCU before proteome experiments were conducted. Therefore, we assumed that this is a very conservative length of time to allow recovery from the chemical treatment. Prior to proteomic experiments, symbiotic state was again confirmed by fluorescence microscopy, chloroplast 23S DNA PCR amplification, and Sanger sequencing.

### Experimental Design for Sampling Anemones Grown Under Different Symbiotic States

Following 1 year of similar culture conditions, anemones of the three symbiotic states (aposymbiotic, *D. trenchii* hosting, and *S. linucheae* hosting) were transferred to six-well culture plates containing 10 mL of autoclaved ASW. Each plate contained five anemones (one per well) ranging in size from 5 to 10 mm that together represented a single biological replicate for its respective treatment. Six biological replicates (each consisting of one plate) were used for each treatment. All culture plates were maintained under the same environmental condition for 7 days before sampling for the comparative molecular study. The plates were partially immersed in a temperature controlled (26°C) water bath and received ∼80 μmol m^–2^s^–1^ of light for 12 h daily (see culture conditions of *Exaiptasia pallida*). Anemones were fed immediately after their initial transfer to new culture plates and their water was replaced with autoclaved ASW 12 h after feeding. Following this, anemones were not fed to avoid detection of *A. franciscana* nauplii proteins in the experimental analyses.

Every anemone treatment group was sampled at two different times: one in the middle of the day (12 p.m.), and the other in the middle of the night (12 a.m.). For each sampling event, five anemones from each treatment groups were pooled into a cryogenic tube serving as technical replicates, forming together one biological replicate. This was repeated in triplicate (*n* = 3) for each treatment group sampled in the day and again at night. The tubes were then centrifuged for 1-min at 78 × *g*. Liquid was removed from the tubes and samples were frozen in liquid nitrogen.

### Symbiont Density Assessment

To extract intact symbiont cells and total proteins from anemones, five anemones colonized by each species of symbiont were individually placed in 1.5 mL tubes and excess seawater was removed. Anemones were next washed with 500 μL PBS, centrifuged for 1 min at 10,000 × *g*, and PBS was removed. Next, 500 μL of PBS with 1% β-mercaptoethanol was added to each anemone which were thoroughly ground in their 1.5 mL tubes with a miniature plastic pestle on ice. The anemone macerates were thoroughly vortexed immediately before a 200 μL aliquot was taken for symbiont cell counts. Symbiont cell counts were performed by counting five 0.1 μL grids on a Neubauer hemocytometer for each of six separate hemocytometer loadings. These were averaged and used to calculate symbiont cells per mL of macerate. The remaining 300 μL of anemone macerate were centrifuged for 5 min at 24,000 × *g* to clear host cell debris and intact symbiont cells from the solution. Two hundred microliters of clear supernatant were aliquoted to a new 1.5 mL tube. Total protein per mL were quantified in triplicate for each sample using the Bradford method with a BSA standard curve modified for use with a plate reader (Bradford, 1976). Finally, symbiont cells/mL macerate were divided by μg host protein/mL macerate for each respective sample, yielding symbiont cells/μg host protein.

### Proteomic and Functional Analyses

Total host protein was extracted and proteomic analyses were performed by Applied Biomics (Hayward, CA, United States) using two-dimensional difference gel electrophoresis (2D DIGE) gels, which allowed for the detection of differential protein expression. The analyses included nine pairwise comparisons: amongst symbiont identities during the day; amongst symbiont identities during night; and between day and night within each symbiont identity (Figure 1B). The 2D DIGE gels were scanned using a Typhoon image scanner (GE Healthcare) and the images were analyzed using Image Quant software (GE-Healthcare). Differential expression ratios of protein were quantified from gel analysis and a paired *t*-test was performed by DeCyder software version 6.5 with a Benjamini-Hochberg procedure to control false discovery rate (FDR) and correct for multiple tests. Of the differentially expressed protein spots detected on the gels, 115 were selected for MALDI-TOF mass spectroscopy using a 5800 System mass spectrometer (AB Sciex). The selection of these proteins was based on highly significant (defined as proteins with FDR corrected *p*-value < 0.005) differential expression. An additional 19 spots were also selected for mass spectrometry identification based on their large expression ratios (>3x relative expression) or their differential expression (defined as FDR corrected *p*-value < 0.05) in four or more of the nine comparisons. Proteins were identified by a genomic database for *E. pallida* (Pringle Lab, Stanford University) using the peptide mass and fragmentation spectra along with the MASCOT search engine (Matrix Science). Significant candidates had either protein score C.I.% or ion C.I.% greater than 95.

Protein sequences were searched against the non-redundant (nr) NCBI database using Blast2GO (Conesa et al., 2005). Gene ontology (GO) terms were obtained and used to derive function. For protein sequences without associated GO terms, function was identified using the Pfam database (Bateman et al., 2002; version 31.0).

## Author Contributions

MR-L conceived the study. MR-L, DM, and AB planned the experimental design. EM conducted the study with the assistance of DM and AB. EM and MR-L conducted the analyses and interpretations, with contributions from DM and AB. EM and MR-L wrote the manuscript. DM and AB edited and reviewed the manuscript.

## Conflict of Interest Statement

The authors declare that the research was conducted in the absence of any commercial or financial relationships that could be construed as a potential conflict of interest.
