# Supplementary material for: Proteomic Basis of Symbiosis: A Heterologous Partner Fails to Duplicate Homologous Colonization in a Novel Cnidarian– Symbiodiniaceae Mutualism
Source: Front Microbiol. 2019 May 31;10:1153. doi: 10.3389/fmicb.2019.01153 (PMC6554683; doi:10.3389/fmicb.2019.01153)
Supplement: Supplementary file 1 [file Table_1.DOCX]

**Supplemental Information Article**

**Title:** Proteomic Basis of Symbiosis: A Heterologous Partner Fails to Duplicate Homologous Success in a Novel Cnidarian- Symbiodiniaceae Mutualism.

**Authors:** Emmanuel Medrano, Daniel G. Merselis, Anthony J. Bellantuono, and Mauricio Rodriguez-Lanetty

The following Supporting Information is provided along the main manuscript:

**Table S1.** List of host proteins represented in the Venn diagrams displayed in Figure 4 of the manuscript.

**Table S2:** List of host proteins in the Venn diagrams showing overlap of differentially expressed proteins within each group of anemones between day and night in figure 5 of the manuscript.

_____________

**Table S1.** List of host proteins represented in the Venn diagrams displayed in Figure 4 of the manuscript. These proteins were either up-regulated or down-regulated in Symbiodiniaceae-colonized anemones when compared to aposymbiotic anemones.

**Proteins upregulated when compared with aposymbiotic anemones (day) – represented in Figure 4A**

| Gel ID | Proteins from anemones colonized by *S. linucheae* | Expression ratio |
| --- | --- | --- |
| 2 | Myosin heavy chain, striated muscle [Exaiptasia pallida] | 1.23 |
| 3 | Endoplasmin [Exaiptasia pallida] | 1.41 |
| 4 | Spectrin alpha chain, non-erythrocytic 1 [Exaiptasia pallida] | 1.16 |
| 7 | Myosin heavy chain, striated muscle [Exaiptasia pallida] | 2.19 |
| 10 | Collagen alpha-5(VI) chain [Exaiptasia pallida] | 1.81 |
| 11 | Calpain-B [Exaiptasia pallida] | 1.52 |
| 18 | Protein disulfide-isomerase [Exaiptasia pallida] | 1.24 |
| 22 | Protein phosphatase 1B [Exaiptasia pallida] | 1.5 |
| 24 | ATP synthase subunit beta, mitochondrial [Exaiptasia pallida] | 3.38 |
| 25 | Tubulin beta chain [Exaiptasia pallida] | 2.25 |
| 33 | Phenylalanine--tRNA ligase alpha subunit [Exaiptasia pallida] | 1.72 |
| 34 | Tubulin alpha-1C chain [Exaiptasia pallida] | 4.44 |
| 35 | hypothetical protein AC249_AIPGENE12332 [Exaiptasia pallida] | 1.76 |
| 39 | Glutamate dehydrogenase [Exaiptasia pallida] | 1.99 |
| 46 | Actin, cytoplasmic [Exaiptasia pallida] | 1.58 |
| 47 | Actin, cytoplasmic [Exaiptasia pallida] | 1.4 |
| 48 | Major antigen [Exaiptasia pallida] | 1.47 |
| 52 | Elongation factor 2 [Exaiptasia pallida] | 2.04 |
| 53 | Pancreatic triacylglycerol lipase [Exaiptasia pallida] | 2.21 |
| 75 | Translationally-controlled tumor protein-like [Exaiptasia pallida] | 1.52 |
| 76 | 14-3-3-like protein 2 [Exaiptasia pallida] | 1.62 |
| 77 | 14-3-3 protein zeta [Exaiptasia pallida] | 1.46 |
| 78 | Peptidyl-prolyl cis-trans isomerase FKBP14 [Exaiptasia pallida] | 1.37 |
| 80 | Rho GDP-dissociation inhibitor 1 [Exaiptasia pallida] | 1.75 |
| 83 | Actin, cytoplasmic [Exaiptasia pallida] | 3.94 |
| 87 | hypothetical protein AC249_AIPGENE8665 [Exaiptasia pallida] | 1.83 |
| 90 | GTPase KRas [Exaiptasia pallida] | 3.32 |
| 94 | Major antigen [Exaiptasia pallida] | 1.79 |
| 111 | Universal stress protein Sll1388 [Exaiptasia pallida] | 1.66 |
| 114 | ADP-ribosylation factor 1 [Exaiptasia pallida] | 1.61 |
| 119 | hypothetical protein AC249_AIPGENE3884 [Exaiptasia pallida] | 1.41 |
| 127 | hypothetical protein AC249_AIPGENE3877 [Exaiptasia pallida] | 8.65 |
| 130 | Calpain-B [Exaiptasia pallida] | 14.93 |
| 131 | Muscle LIM protein Mlp84B [Exaiptasia pallida] | 1.2 |
| 132 | hypothetical protein AC249_AIPGENE7839 [Exaiptasia pallida] | 1.71 |
| 133 | Actin, cytoplasmic [Exaiptasia pallida] | 5.56 |
| 137 | Major antigen [Exaiptasia pallida] | 3.5 |
| 140 | E3 ubiquitin-protein ligase DZIP3 [Exaiptasia pallida] | 1.17 |

| Gel ID | Proteins from anemones colonized by *D. trenchii* | Expression ratio |
| --- | --- | --- |
| 49 | Actin, cytoplasmic [Exaiptasia pallida] | 1.22 |
| 57 | Isocitrate dehydrogenase [NAD] subunit 1, mitochondrial [Exaiptasia pallida] | 2.26 |
| 64 | Actin, cytoplasmic [Exaiptasia pallida] | 1.43 |
| 67 | Actin, cytoplasmic [Exaiptasia pallida] | 9.66 |
| 70 | Phosphoethanolamine N-methyltransferase 3 [Exaiptasia pallida] | 1.41 |
| 72 | Plasma kallikrein [Exaiptasia pallida] | 2.89 |
| 97 | Programmed cell death protein 6 [Exaiptasia pallida] | 6.37 |
| 113 | Sorcin [Exaiptasia pallida] | 1.53 |
| 118 | hypothetical protein AC249_AIPGENE10882 [Exaiptasia pallida] | 1.36 |

| Gel ID | Proteins shared | Expression in *S. linucheae-*colonized anemones | Expression in *D. trenchii-*colonized anemones |
| --- | --- | --- | --- |
| 20 | Failed axon connections-like [Exaiptasia pallida] | 1.07 | 1.19 |
| 23 | Failed axon connections-like [Exaiptasia pallida] | 2.23 | 1.62 |
| 40 | Glutamate dehydrogenase [Exaiptasia pallida] | 1.54 | 1.84 |
| 42 | Gelsolin-like protein 2 [Exaiptasia pallida] | 1.41 | 1.48 |
| 43 | Gelsolin-like protein 1 [Exaiptasia pallida] | 1.14 | 1.65 |
| 68 | Actin, cytoplasmic [Exaiptasia pallida] | 1.95 | 1.07 |
| 91 | Actin, cytoplasmic 1 [Exaiptasia pallida] | 1.89 | 2.83 |
| 106 | Myosin regulatory light chain 12B [Exaiptasia pallida] | 2.74 | 4.11 |
| 129 | Protein NPC2-like [Exaiptasia pallida] | 2.21 | 3.96 |

**Proteins upregulated when compared with aposymbiotic anemones (night) – represented in Figure 4B**

| Gel ID | Proteins from anemones colonized by *S. linucheae* | Expression Ratio |
| --- | --- | --- |
| 4 | Spectrin alpha chain, non-erythrocytic 1 [Exaiptasia pallida] | 1.39 |
| 7 | Myosin heavy chain, striated muscle [Exaiptasia pallida] | 3.29 |
| 10 | Collagen alpha-5(VI) chain [Exaiptasia pallida] | 1.94 |
| 16 | hypothetical protein AC249_AIPGENE16868 [Exaiptasia pallida] | 1.35 |
| 22 | Protein phosphatase 1B [Exaiptasia pallida] | 1.41 |
| 25 | Tubulin beta chain [Exaiptasia pallida] | 2.21 |
| 34 | Tubulin alpha-1C chain [Exaiptasia pallida] | 3.27 |
| 35 | hypothetical protein AC249_AIPGENE12332 [Exaiptasia pallida] | 2.21 |
| 52 | Elongation factor 2 [Exaiptasia pallida] | 2.16 |
| 53 | Pancreatic triacylglycerol lipase [Exaiptasia pallida] | 2.11 |
| 78 | Peptidyl-prolyl cis-trans isomerase FKBP14 [Exaiptasia pallida] | 1.27 |
| 87 | hypothetical protein AC249_AIPGENE8665 [Exaiptasia pallida] | 1.43 |
| 91 | Actin, cytoplasmic 1 [Exaiptasia pallida] | 4.29 |
| 92 | Golgi-associated plant pathogenesis-related protein 1 [Exaiptasia pallida] | 1.84 |
| 93 | Sorcin [Exaiptasia pallida] | 2.23 |
| 111 | Universal stress protein Sll1388 [Exaiptasia pallida] | 1.67 |
| 114 | ADP-ribosylation factor 1 [Exaiptasia pallida] | 1.64 |
| 127 | hypothetical protein AC249_AIPGENE3877 [Exaiptasia pallida] | 4.72 |
| 129 | Protein NPC2-like [Exaiptasia pallida] | 6.56 |
| 130 | Calpain-B [Exaiptasia pallida] | 11.62 |
| 131 | Muscle LIM protein Mlp84B [Exaiptasia pallida] | 1.25 |
| 132 | hypothetical protein AC249_AIPGENE7839 [Exaiptasia pallida] | 1.62 |
| 133 | Actin, cytoplasmic [Exaiptasia pallida] | 6.62 |
| 137 | Major antigen [Exaiptasia pallida] | 3.41 |

| Gel ID | Proteins from anemones colonized by *D. trenchii* | Expression Ratio |
| --- | --- | --- |
| 20 | Failed axon connections-like [Exaiptasia pallida] | 1.54 |
| 23 | Failed axon connections-like [Exaiptasia pallida] | 2.12 |
| 67 | Actin, cytoplasmic [Exaiptasia pallida] | 7.54 |
| 68 | Actin, cytoplasmic [Exaiptasia pallida] | 1.42 |
| 72 | Plasma kallikrein [Exaiptasia pallida] | 2.2 |
| 97 | Programmed cell death protein 6 [Exaiptasia pallida] | 3.55 |

| Gel ID | Proteins shared | Expression in *S. linucheae*-colonized anemones | Expression in *D. trenchii-* colonized anemones |
| --- | --- | --- | --- |
| 2 | Myosin heavy chain, striated muscle [Exaiptasia pallida] | 1.41 | 1.08 |
| 3 | Endoplasmin [Exaiptasia pallida] | 1.44 | 1.27 |
| 11 | Calpain-B [Exaiptasia pallida] | 1.42 | 1.3 |
| 18 | Protein disulfide-isomerase [Exaiptasia pallida] | 1.32 | 1.16 |
| 24 | ATP synthase subunit beta, mitochondrial [Exaiptasia pallida] | 3.39 | 1.72 |
| 33 | Phenylalanine--tRNA ligase alpha subunit [Exaiptasia pallida] | 1.5 | 1.43 |
| 39 | Glutamate dehydrogenase [Exaiptasia pallida] | 2.55 | 1.61 |
| 40 | Glutamate dehydrogenase [Exaiptasia pallida] | 2.22 | 1.49 |
| 42 | Gelsolin-like protein 2 [Exaiptasia pallida] | 1.37 | 1.52 |
| 43 | Gelsolin-like protein 1 [Exaiptasia pallida] | 1.65 | 1.33 |
| 46 | Actin, cytoplasmic [Exaiptasia pallida] | 1.68 | 1.34 |
| 47 | Actin, cytoplasmic [Exaiptasia pallida] | 1.6 | 1.29 |
| 48 | Major antigen [Exaiptasia pallida] | 1.66 | 1.49 |
| 70 | Phosphoethanolamine N-methyltransferase 3 [Exaiptasia pallida] | 1.34 | 1.91 |
| 75 | Translationally-controlled tumor protein-like [Exaiptasia pallida] | 1.57 | 1.21 |
| 76 | 14-3-3-like protein 2 [Exaiptasia pallida] | 1.94 | 1.49 |
| 77 | 14-3-3 protein zeta [Exaiptasia pallida] | 1.39 | 1.33 |
| 80 | Rho GDP-dissociation inhibitor 1 [Exaiptasia pallida] | 1.37 | 1.51 |
| 90 | GTPase KRas [Exaiptasia pallida] | 2.47 | 1.78 |
| 94 | Major antigen [Exaiptasia pallida] | 1.58 | 1.61 |
| 106 | Myosin regulatory light chain 12B [Exaiptasia pallida] | 5.55 | 3.14 |
| 140 | E3 ubiquitin-protein ligase DZIP3 [Exaiptasia pallida] | 1.19 | 1.77 |

**Proteins downregulated when compared with aposymbiotic anemones (day) – represented in Figure 4D**

| Gel ID | Proteins from anemones colonized *S. linucheae* | Expression Ratio |
| --- | --- | --- |
| 21 | Lysostaphin [Exaiptasia pallida] | -1.69 |
| 26 | Tubulin alpha-1C chain [Exaiptasia pallida] | -1.57 |
| 31 | Selenium-binding protein 1 [Exaiptasia pallida] | -4.11 |
| 32 | Moesin/ezrin/radixin-like 1 [Exaiptasia pallida] | -1.8 |
| 36 | hypothetical protein AC249_AIPGENE13469 [Exaiptasia pallida] | -1.61 |
| 49 | Actin, cytoplasmic [Exaiptasia pallida] | -1.65 |
| 50 | Actin, cytoplasmic [Exaiptasia pallida] | -1.5 |
| 51 | Actin, cytoplasmic [Exaiptasia pallida] | -1.27 |
| 54 | Serine--pyruvate aminotransferase, mitochondrial [Exaiptasia pallida] | -1.64 |
| 55 | Fructose-bisphosphate aldolase C [Exaiptasia pallida] | -1.63 |
| 56 | Serine--pyruvate aminotransferase, mitochondrial [Exaiptasia pallida] | -1.79 |
| 60 | hypothetical protein AC249_AIPGENE28714 [Exaiptasia pallida] | -1.84 |
| 61 | Fumarylacetoacetate hydrolase domain-containing protein 2 [Exaiptasia pallida] | -1.85 |
| 64 | Actin, cytoplasmic [Exaiptasia pallida] | -1.68 |
| 82 | Actin, cytoplasmic [Exaiptasia pallida] | -3.11 |
| 113 | Sorcin [Exaiptasia pallida] | -1.67 |
| 118 | hypothetical protein AC249_AIPGENE10882 [Exaiptasia pallida] | -1.88 |
| 126 | Myosin regulatory light chain sqh [Exaiptasia pallida] | -2.02 |
| 128 | Rho GDP-dissociation inhibitor 1 [Exaiptasia pallida] | -1.48 |
| 141 | hypothetical protein AC249_AIPGENE3818 [Exaiptasia pallida] | -1.56 |
| 144 | SH3 domain-binding glutamic acid-rich-like protein 3 [Exaiptasia pallida] | -2.43 |
| 146 | Small nuclear ribonucleoprotein G [Exaiptasia pallida] | -2.25 |

| Gel ID | Proteins from anemones colonized by *D. trenchii* | Expression Ratio |
| --- | --- | --- |
| 2 | Myosin heavy chain, striated muscle [Exaiptasia pallida] | -1.39 |
| 3 | Endoplasmin [Exaiptasia pallida] | -1.18 |
| 4 | Spectrin alpha chain, non-erythrocytic 1 [Exaiptasia pallida] | -1.46 |
| 12 | Calpain-B [Exaiptasia pallida] | -1.48 |
| 18 | Protein disulfide-isomerase [Exaiptasia pallida] | -1.23 |
| 59 | Malate dehydrogenase, cytoplasmic [Exaiptasia pallida] | -1.82 |
| 78 | Peptidyl-prolyl cis-trans isomerase FKBP14 [Exaiptasia pallida] | -1.14 |
| 92 | Golgi-associated plant pathogenesis-related protein 1 [Exaiptasia pallida] | -2.69 |
| 93 | Sorcin [Exaiptasia pallida] | -3.09 |
| 94 | Major antigen [Exaiptasia pallida] | -1.48 |
| 110 | Actin, cytoplasmic [Exaiptasia pallida] | -2.31 |
| 119 | hypothetical protein AC249_AIPGENE3884 [Exaiptasia pallida] | -1.3 |
| 125 | Myophilin [Exaiptasia pallida] | -1.4 |
| 132 | hypothetical protein AC249_AIPGENE7839 [Exaiptasia pallida] | -1.68 |
| 136 | Myotrophin [Exaiptasia pallida] | -1.63 |

| Gel ID | Proteins shared | Expression in *S. linucheae-* colonized anemones | Expression in *D. trenchii-* colonized anemones |
| --- | --- | --- | --- |
| 1 | Myosin heavy chain, striated muscle [Exaiptasia pallida] | -1.16 | -2.06 |
| 8 | hypothetical protein AC249_AIPGENE16477 [Exaiptasia pallida] | -1.86 | -1.9 |
| 13 | Phosphoenolpyruvate carboxykinase [GTP], mitochondrial [Exaiptasia pallida] | -1.32 | -1.65 |
| 19 | Neuronal pentraxin-2 [Exaiptasia pallida] | -2.46 | -1.96 |
| 37 | Calpain-B [Exaiptasia pallida] | -1.81 | -1.48 |
| 38 | Alpha-enolase [Exaiptasia pallida] | -1.45 | -1.35 |
| 44 | Fumarylacetoacetase [Exaiptasia pallida] | -1.91 | -1.49 |
| 45 | Heat shock cognate 71 kDa protein [Exaiptasia pallida] | -1.69 | -1.66 |
| 69 | Inactive pancreatic lipase-related protein 1 [Exaiptasia pallida] | -1.58 | -1.87 |
| 79 | 14-3-3 protein zeta [Exaiptasia pallida] | -1.58 | -1.45 |
| 84 | S-crystallin 4 [Exaiptasia pallida] | -1.67 | -1.26 |
| 112 | ADP-ribosylation factor 1 [Exaiptasia pallida] | -1.86 | -1.56 |
| 121 | Ectin [Exaiptasia pallida] | -1.17 | -1.83 |
| 122 | hypothetical protein AC249_AIPGENE3884 [Exaiptasia pallida] | -1.48 | -1.15 |

**Proteins downregulated when compared with aposymbiotic anemones (night) – represented in Figure 4E**

| Gel ID | Proteins from anemones colonized by *S. linucheae* | Expression Ratio |
| --- | --- | --- |
| 32 | Moesin/ezrin/radixin-like 1 [Exaiptasia pallida] | -2.3 |
| 49 | Actin, cytoplasmic [Exaiptasia pallida] | -1.69 |
| 54 | Serine--pyruvate aminotransferase, mitochondrial [Exaiptasia pallida] | -1.47 |
| 60 | hypothetical protein AC249_AIPGENE28714 [Exaiptasia pallida] | -1.41 |
| 61 | Fumarylacetoacetate hydrolase domain-containing protein 2 [Exaiptasia pallida] | -1.66 |
| 64 | Actin, cytoplasmic [Exaiptasia pallida] | -1.72 |
| 84 | S-crystallin 4 [Exaiptasia pallida] | -1.6 |
| 118 | hypothetical protein AC249_AIPGENE10882 [Exaiptasia pallida] | -2.21 |
| 141 | hypothetical protein AC249_AIPGENE3818 [Exaiptasia pallida] | -1.43 |
| 144 | SH3 domain-binding glutamic acid-rich-like protein 3 [Exaiptasia pallida] | -1.82 |
| 146 | Small nuclear ribonucleoprotein G [Exaiptasia pallida] | -2.24 |

| Gel ID | Proteins from anemones colonized by *D. trenchii* | Expression Ratio |
| --- | --- | --- |
| 1 | Myosin heavy chain, striated muscle [Exaiptasia pallida] | -1.17 |
| 16 | hypothetical protein AC249_AIPGENE16868 [Exaiptasia pallida] | -1.29 |
| 45 | Heat shock cognate 71 kDa protein [Exaiptasia pallida] | -1.42 |
| 55 | Fructose-bisphosphate aldolase C [Exaiptasia pallida] | -1.53 |
| 69 | Inactive pancreatic lipase-related protein 1 [Exaiptasia pallida] | -1.73 |
| 92 | Golgi-associated plant pathogenesis-related protein 1 [Exaiptasia pallida] | -2.3 |
| 93 | Sorcin [Exaiptasia pallida] | -3.03 |
| 110 | Actin, cytoplasmic [Exaiptasia pallida] | -2.3 |
| 119 | hypothetical protein AC249_AIPGENE3884 [Exaiptasia pallida] | -1.54 |
| 121 | Ectin [Exaiptasia pallida] | -1.81 |

| Gel ID | Proteins shared | Expression in *S. linucheae-* colonized anemones | Expression in *D. trenchii-* colonized  anemones |
| --- | --- | --- | --- |
| 8 | hypothetical protein AC249_AIPGENE16477 [Exaiptasia pallida] | -1.49 | -1.47 |
| 13 | Phosphoenolpyruvate carboxykinase [GTP], mitochondrial [Exaiptasia pallida] | -1.37 | -1.66 |
| 19 | Neuronal pentraxin-2 [Exaiptasia pallida] | -2.02 | -1.86 |
| 21 | Lysostaphin [Exaiptasia pallida] | -1.71 | -1.44 |
| 26 | Tubulin alpha-1C chain [Exaiptasia pallida] | -1.5 | -1.35 |
| 31 | Selenium-binding protein 1 [Exaiptasia pallida] | -4.24 | -1.35 |
| 36 | hypothetical protein AC249_AIPGENE13469 [Exaiptasia pallida] | -1.75 | -1.21 |
| 37 | Calpain-B [Exaiptasia pallida] | -1.57 | -1.77 |
| 38 | Alpha-enolase [Exaiptasia pallida] | -1.53 | -1.54 |
| 44 | Fumarylacetoacetase [Exaiptasia pallida] | -2.02 | -1.58 |
| 50 | Actin, cytoplasmic [Exaiptasia pallida] | -1.69 | -1.23 |
| 56 | Serine--pyruvate aminotransferase, mitochondrial [Exaiptasia pallida] | -2.26 | -1.38 |
| 59 | Malate dehydrogenase, cytoplasmic [Exaiptasia pallida] | -1.13 | -1.42 |
| 79 | 14-3-3 protein zeta [Exaiptasia pallida] | -1.51 | -1.5 |
| 82 | Actin, cytoplasmic [Exaiptasia pallida] | -1.79 | -1.25 |
| 112 | ADP-ribosylation factor 1 [Exaiptasia pallida] | -1.61 | -1.32 |
| 122 | hypothetical protein AC249_AIPGENE3884 [Exaiptasia pallida] | -1.65 | -1.29 |
| 128 | Rho GDP-dissociation inhibitor 1 [Exaiptasia pallida] | -1.26 | -1.15 |

_______________

**Table S2:** List of host proteins in the Venn diagrams showing overlap of differentially expressed proteins within each group of anemones between day and night in figure 5 of the manuscript.

**Proteins upregulated in anemones colonized by *S. linucheae* during the day represented in Figure 5A**

| Day | | |
| --- | --- | --- |
| Gel ID | Protein Identitiy | Expression ratio |
| 2 | Myosin heavy chain, striated muscle [Exaiptasia pallida] | 1.23 |
| 3 | Endoplasmin [Exaiptasia pallida] | 1.41 |
| 4 | Spectrin alpha chain, non-erythrocytic 1 [Exaiptasia pallida] | 1.16 |
| 7 | Myosin heavy chain, striated muscle [Exaiptasia pallida] | 2.19 |
| 10 | Collagen alpha-5(VI) chain [Exaiptasia pallida] | 1.81 |
| 11 | Calpain-B [Exaiptasia pallida] | 1.52 |
| 18 | Protein disulfide-isomerase [Exaiptasia pallida] | 1.24 |
| 20 | Failed axon connections-like [Exaiptasia pallida] | 1.07 |
| 22 | Protein phosphatase 1B [Exaiptasia pallida] | 1.5 |
| 23 | Failed axon connections-like [Exaiptasia pallida] | 2.23 |
| 24 | ATP synthase subunit beta, mitochondrial [Exaiptasia pallida] | 3.38 |
| 25 | Tubulin beta chain [Exaiptasia pallida] | 2.25 |
| 33 | Phenylalanine--tRNA ligase alpha subunit [Exaiptasia pallida] | 1.72 |
| 34 | Tubulin alpha-1C chain [Exaiptasia pallida] | 4.44 |
| 35 | hypothetical protein AC249_AIPGENE12332 [Exaiptasia pallida] | 1.76 |
| 39 | Glutamate dehydrogenase [Exaiptasia pallida] | 1.99 |
| 40 | Glutamate dehydrogenase [Exaiptasia pallida] | 1.54 |
| 42 | Gelsolin-like protein 2 [Exaiptasia pallida] | 1.41 |
| 43 | Gelsolin-like protein 1 [Exaiptasia pallida] | 1.14 |
| 46 | Actin, cytoplasmic [Exaiptasia pallida] | 1.58 |
| 47 | Actin, cytoplasmic [Exaiptasia pallida] | 1.4 |
| 48 | Major antigen [Exaiptasia pallida] | 1.47 |
| 52 | Elongation factor 2 [Exaiptasia pallida] | 2.04 |
| 53 | Pancreatic triacylglycerol lipase [Exaiptasia pallida] | 2.21 |
| 68 | Actin, cytoplasmic [Exaiptasia pallida] | 1.95 |
| 75 | Translationally-controlled tumor protein-like [Exaiptasia pallida] | 1.52 |
| 76 | 14-3-3-like protein 2 [Exaiptasia pallida] | 1.62 |
| 77 | 14-3-3 protein zeta [Exaiptasia pallida] | 1.46 |
| 78 | Peptidyl-prolyl cis-trans isomerase FKBP14 [Exaiptasia pallida] | 1.37 |
| 80 | Rho GDP-dissociation inhibitor 1 [Exaiptasia pallida] | 1.75 |
| 83 | Actin, cytoplasmic [Exaiptasia pallida] | 3.94 |
| 87 | hypothetical protein AC249_AIPGENE8665 [Exaiptasia pallida] | 1.83 |
| 90 | GTPase KRas [Exaiptasia pallida] | 3.32 |
| 91 | Actin, cytoplasmic 1 [Exaiptasia pallida] | 1.89 |
| 94 | Major antigen [Exaiptasia pallida] | 1.79 |
| 106 | Myosin regulatory light chain 12B [Exaiptasia pallida] | 2.74 |
| 111 | Universal stress protein Sll1388 [Exaiptasia pallida] | 1.66 |
| 114 | ADP-ribosylation factor 1 [Exaiptasia pallida] | 1.61 |
| 119 | hypothetical protein AC249_AIPGENE3884 [Exaiptasia pallida] | 1.41 |
| 127 | hypothetical protein AC249_AIPGENE3877 [Exaiptasia pallida] | 8.65 |
| 129 | Protein NPC2-like [Exaiptasia pallida] | 2.21 |
| 130 | Calpain-B [Exaiptasia pallida] | 14.93 |
| 131 | Muscle LIM protein Mlp84B [Exaiptasia pallida] | 1.2 |
| 132 | hypothetical protein AC249_AIPGENE7839 [Exaiptasia pallida] | 1.71 |
| 133 | Actin, cytoplasmic [Exaiptasia pallida] | 5.56 |
| 137 | Major antigen [Exaiptasia pallida] | 3.5 |
| 140 | E3 ubiquitin-protein ligase DZIP3 [Exaiptasia pallida] | 1.17 |

**Proteins upregulated in anemones colonized by *S. linucheae* during the night represented in Figure 5A**

| Night | | |
| --- | --- | --- |
| Gel ID | Protein Identity | Expression Ratio |
| 2 | Myosin heavy chain, striated muscle [Exaiptasia pallida] | 1.41 |
| 3 | Endoplasmin [Exaiptasia pallida] | 1.44 |
| 4 | Spectrin alpha chain, non-erythrocytic 1 [Exaiptasia pallida] | 1.39 |
| 7 | Myosin heavy chain, striated muscle [Exaiptasia pallida] | 3.29 |
| 10 | Collagen alpha-5(VI) chain [Exaiptasia pallida] | 1.94 |
| 11 | Calpain-B [Exaiptasia pallida] | 1.42 |
| 16 | hypothetical protein AC249_AIPGENE16868 [Exaiptasia pallida] | 1.35 |
| 18 | Protein disulfide-isomerase [Exaiptasia pallida] | 1.32 |
| 22 | Protein phosphatase 1B [Exaiptasia pallida] | 1.41 |
| 24 | ATP synthase subunit beta, mitochondrial [Exaiptasia pallida] | 3.39 |
| 25 | Tubulin beta chain [Exaiptasia pallida] | 2.21 |
| 33 | Phenylalanine--tRNA ligase alpha subunit [Exaiptasia pallida] | 1.5 |
| 34 | Tubulin alpha-1C chain [Exaiptasia pallida] | 3.27 |
| 35 | hypothetical protein AC249_AIPGENE12332 [Exaiptasia pallida] | 2.21 |
| 39 | Glutamate dehydrogenase [Exaiptasia pallida] | 2.55 |
| 40 | Glutamate dehydrogenase [Exaiptasia pallida] | 2.22 |
| 42 | Gelsolin-like protein 2 [Exaiptasia pallida] | 1.37 |
| 43 | Gelsolin-like protein 1 [Exaiptasia pallida] | 1.65 |
| 46 | Actin, cytoplasmic [Exaiptasia pallida] | 1.68 |
| 47 | Actin, cytoplasmic [Exaiptasia pallida] | 1.6 |
| 48 | Major antigen [Exaiptasia pallida] | 1.66 |
| 52 | Elongation factor 2 [Exaiptasia pallida] | 2.16 |
| 53 | Pancreatic triacylglycerol lipase [Exaiptasia pallida] | 2.11 |
| 70 | Phosphoethanolamine N-methyltransferase 3 [Exaiptasia pallida] | 1.34 |
| 75 | Translationally-controlled tumor protein-like [Exaiptasia pallida] | 1.57 |
| 76 | 14-3-3-like protein 2 [Exaiptasia pallida] | 1.94 |
| 77 | 14-3-3 protein zeta [Exaiptasia pallida] | 1.39 |
| 78 | Peptidyl-prolyl cis-trans isomerase FKBP14 [Exaiptasia pallida] | 1.27 |
| 80 | Rho GDP-dissociation inhibitor 1 [Exaiptasia pallida] | 1.37 |
| 87 | hypothetical protein AC249_AIPGENE8665 [Exaiptasia pallida] | 1.43 |
| 90 | GTPase KRas [Exaiptasia pallida] | 2.47 |
| 91 | Actin, cytoplasmic 1 [Exaiptasia pallida] | 4.29 |
| 92 | Golgi-associated plant pathogenesis-related protein 1 [Exaiptasia pallida] | 1.84 |
| 93 | Sorcin [Exaiptasia pallida] | 2.23 |
| 94 | Major antigen [Exaiptasia pallida] | 1.58 |
| 106 | Myosin regulatory light chain 12B [Exaiptasia pallida] | 5.55 |
| 111 | Universal stress protein Sll1388 [Exaiptasia pallida] | 1.67 |
| 114 | ADP-ribosylation factor 1 [Exaiptasia pallida] | 1.64 |
| 127 | hypothetical protein AC249_AIPGENE3877 [Exaiptasia pallida] | 4.72 |
| 129 | Protein NPC2-like [Exaiptasia pallida] | 6.56 |
| 130 | Calpain-B [Exaiptasia pallida] | 11.62 |
| 131 | Muscle LIM protein Mlp84B [Exaiptasia pallida] | 1.25 |
| 132 | hypothetical protein AC249_AIPGENE7839 [Exaiptasia pallida] | 1.62 |
| 133 | Actin, cytoplasmic [Exaiptasia pallida] | 6.62 |
| 137 | Major antigen [Exaiptasia pallida] | 3.41 |
| 140 | E3 ubiquitin-protein ligase DZIP3 [Exaiptasia pallida] | 1.19 |

**Proteins downregulated in anemones colonized by *S. linucheae* during the day represented in Figure 5B**

| Day | | |
| --- | --- | --- |
| Gel ID | Protein Identity | Expression Ratio |
| 1 | Myosin heavy chain, striated muscle [Exaiptasia pallida] | -1.16 |
| 8 | hypothetical protein AC249_AIPGENE16477 [Exaiptasia pallida] | -1.86 |
| 13 | Phosphoenolpyruvate carboxykinase [GTP], mitochondrial [Exaiptasia pallida] | -1.32 |
| 19 | Neuronal pentraxin-2 [Exaiptasia pallida] | -2.46 |
| 21 | Lysostaphin [Exaiptasia pallida] | -1.69 |
| 26 | Tubulin alpha-1C chain [Exaiptasia pallida] | -1.57 |
| 31 | Selenium-binding protein 1 [Exaiptasia pallida] | -4.11 |
| 32 | Moesin/ezrin/radixin-like 1 [Exaiptasia pallida] | -1.8 |
| 36 | hypothetical protein AC249_AIPGENE13469 [Exaiptasia pallida] | -1.61 |
| 37 | Calpain-B [Exaiptasia pallida] | -1.81 |
| 38 | Alpha-enolase [Exaiptasia pallida] | -1.45 |
| 44 | Fumarylacetoacetase [Exaiptasia pallida] | -1.91 |
| 45 | Heat shock cognate 71 kDa protein [Exaiptasia pallida] | -1.69 |
| 49 | Actin, cytoplasmic [Exaiptasia pallida] | -1.65 |
| 50 | Actin, cytoplasmic [Exaiptasia pallida] | -1.5 |
| 51 | Actin, cytoplasmic [Exaiptasia pallida] | -1.27 |
| 54 | Serine--pyruvate aminotransferase, mitochondrial [Exaiptasia pallida] | -1.64 |
| 55 | Fructose-bisphosphate aldolase C [Exaiptasia pallida] | -1.63 |
| 56 | Serine--pyruvate aminotransferase, mitochondrial [Exaiptasia pallida] | -1.79 |
| 60 | hypothetical protein AC249_AIPGENE28714 [Exaiptasia pallida] | -1.84 |
| 61 | Fumarylacetoacetate hydrolase domain-containing protein 2 [Exaiptasia pallida] | -1.85 |
| 64 | Actin, cytoplasmic [Exaiptasia pallida] | -1.68 |
| 69 | Inactive pancreatic lipase-related protein 1 [Exaiptasia pallida] | -1.58 |
| 79 | 14-3-3 protein zeta [Exaiptasia pallida] | -1.58 |
| 82 | Actin, cytoplasmic [Exaiptasia pallida] | -3.11 |
| 84 | S-crystallin 4 [Exaiptasia pallida] | -1.67 |
| 112 | ADP-ribosylation factor 1 [Exaiptasia pallida] | -1.86 |
| 113 | Sorcin [Exaiptasia pallida] | -1.67 |
| 118 | hypothetical protein AC249_AIPGENE10882 [Exaiptasia pallida] | -1.88 |
| 121 | Ectin [Exaiptasia pallida] | -1.17 |
| 122 | hypothetical protein AC249_AIPGENE3884 [Exaiptasia pallida] | -1.48 |
| 126 | Myosin regulatory light chain sqh [Exaiptasia pallida] | -2.02 |
| 128 | Rho GDP-dissociation inhibitor 1 [Exaiptasia pallida] | -1.48 |
| 141 | hypothetical protein AC249_AIPGENE3818 [Exaiptasia pallida] | -1.56 |
| 144 | SH3 domain-binding glutamic acid-rich-like protein 3 [Exaiptasia pallida] | -2.43 |
| 146 | Small nuclear ribonucleoprotein G [Exaiptasia pallida] | -2.25 |

**Proteins downregulated in anemones colonized by *S. linucheae* during the night represented in Figure 5B**

| Night | | |
| --- | --- | --- |
| Gel ID | Protein Identity | Expression Ratio |
| 8 | hypothetical protein AC249_AIPGENE16477 [Exaiptasia pallida] | -1.49 |
| 13 | Phosphoenolpyruvate carboxykinase [GTP], mitochondrial [Exaiptasia pallida] | -1.37 |
| 19 | Neuronal pentraxin-2 [Exaiptasia pallida] | -2.02 |
| 21 | Lysostaphin [Exaiptasia pallida] | -1.71 |
| 26 | Tubulin alpha-1C chain [Exaiptasia pallida] | -1.5 |
| 31 | Selenium-binding protein 1 [Exaiptasia pallida] | -4.24 |
| 32 | Moesin/ezrin/radixin-like 1 [Exaiptasia pallida] | -2.3 |
| 36 | hypothetical protein AC249_AIPGENE13469 [Exaiptasia pallida] | -1.75 |
| 37 | Calpain-B [Exaiptasia pallida] | -1.57 |
| 38 | Alpha-enolase [Exaiptasia pallida] | -1.53 |
| 44 | Fumarylacetoacetase [Exaiptasia pallida] | -2.02 |
| 49 | Actin, cytoplasmic [Exaiptasia pallida] | -1.69 |
| 50 | Actin, cytoplasmic [Exaiptasia pallida] | -1.69 |
| 54 | Serine--pyruvate aminotransferase, mitochondrial [Exaiptasia pallida] | -1.47 |
| 56 | Serine--pyruvate aminotransferase, mitochondrial [Exaiptasia pallida] | -2.26 |
| 59 | Malate dehydrogenase, cytoplasmic [Exaiptasia pallida] | -1.13 |
| 60 | hypothetical protein AC249_AIPGENE28714 [Exaiptasia pallida] | -1.41 |
| 61 | Fumarylacetoacetate hydrolase domain-containing protein 2 [Exaiptasia pallida] | -1.66 |
| 64 | Actin, cytoplasmic [Exaiptasia pallida] | -1.72 |
| 79 | 14-3-3 protein zeta [Exaiptasia pallida] | -1.51 |
| 82 | Actin, cytoplasmic [Exaiptasia pallida] | -1.79 |
| 84 | S-crystallin 4 [Exaiptasia pallida] | -1.6 |
| 112 | ADP-ribosylation factor 1 [Exaiptasia pallida] | -1.61 |
| 118 | hypothetical protein AC249_AIPGENE10882 [Exaiptasia pallida] | -2.21 |
| 122 | hypothetical protein AC249_AIPGENE3884 [Exaiptasia pallida] | -1.65 |
| 128 | Rho GDP-dissociation inhibitor 1 [Exaiptasia pallida] | -1.26 |
| 141 | hypothetical protein AC249_AIPGENE3818 [Exaiptasia pallida] | -1.43 |
| 144 | SH3 domain-binding glutamic acid-rich-like protein 3 [Exaiptasia pallida] | -1.82 |
| 146 | Small nuclear ribonucleoprotein G [Exaiptasia pallida] | -2.24 |

**Proteins upregulated in anemones colonized by *D. trenchii* during the day represented in Figure 5C**

| Day | | |
| --- | --- | --- |
| Gel ID | Protein Identity | Expression Ratio |
| 20 | Failed axon connections-like [Exaiptasia pallida] | 1.19 |
| 23 | Failed axon connections-like [Exaiptasia pallida] | 1.62 |
| 40 | Glutamate dehydrogenase [Exaiptasia pallida] | 1.84 |
| 42 | Gelsolin-like protein 2 [Exaiptasia pallida] | 1.48 |
| 43 | Gelsolin-like protein 1 [Exaiptasia pallida] | 1.65 |
| 49 | Actin, cytoplasmic [Exaiptasia pallida] | 1.22 |
| 57 | Isocitrate dehydrogenase [NAD] subunit 1, mitochondrial [Exaiptasia pallida] | 2.26 |
| 64 | Actin, cytoplasmic [Exaiptasia pallida] | 1.43 |
| 67 | Actin, cytoplasmic [Exaiptasia pallida] | 9.66 |
| 68 | Actin, cytoplasmic [Exaiptasia pallida] | 1.07 |
| 70 | Phosphoethanolamine N-methyltransferase 3 [Exaiptasia pallida] | 1.41 |
| 72 | Plasma kallikrein [Exaiptasia pallida] | 2.89 |
| 91 | Actin, cytoplasmic 1 [Exaiptasia pallida] | 2.83 |
| 97 | Programmed cell death protein 6 [Exaiptasia pallida] | 6.37 |
| 106 | Myosin regulatory light chain 12B [Exaiptasia pallida] | 4.11 |
| 113 | Sorcin [Exaiptasia pallida] | 1.53 |
| 118 | hypothetical protein AC249_AIPGENE10882 [Exaiptasia pallida] | 1.36 |
| 129 | Protein NPC2-like [Exaiptasia pallida] | 3.96 |

**Proteins upregulated in anemones colonized by *D. trenchii* during the night represented in Figure 5C**

| Night | | |
| --- | --- | --- |
| Gel ID | Protein Identity | Expression Ratio |
| 2 | Myosin heavy chain, striated muscle [Exaiptasia pallida] | 1.08 |
| 3 | Endoplasmin [Exaiptasia pallida] | 1.27 |
| 11 | Calpain-B [Exaiptasia pallida] | 1.3 |
| 18 | Protein disulfide-isomerase [Exaiptasia pallida] | 1.16 |
| 20 | Failed axon connections-like [Exaiptasia pallida] | 1.54 |
| 23 | Failed axon connections-like [Exaiptasia pallida] | 2.12 |
| 24 | ATP synthase subunit beta, mitochondrial [Exaiptasia pallida] | 1.72 |
| 33 | Phenylalanine--tRNA ligase alpha subunit [Exaiptasia pallida] | 1.43 |
| 39 | Glutamate dehydrogenase [Exaiptasia pallida] | 1.61 |
| 40 | Glutamate dehydrogenase [Exaiptasia pallida] | 1.49 |
| 42 | Gelsolin-like protein 2 [Exaiptasia pallida] | 1.52 |
| 43 | Gelsolin-like protein 1 [Exaiptasia pallida] | 1.33 |
| 46 | Actin, cytoplasmic [Exaiptasia pallida] | 1.34 |
| 47 | Actin, cytoplasmic [Exaiptasia pallida] | 1.29 |
| 48 | Major antigen [Exaiptasia pallida] | 1.49 |
| 67 | Actin, cytoplasmic [Exaiptasia pallida] | 7.54 |
| 68 | Actin, cytoplasmic [Exaiptasia pallida] | 1.42 |
| 70 | Phosphoethanolamine N-methyltransferase 3 [Exaiptasia pallida] | 1.91 |
| 72 | Plasma kallikrein [Exaiptasia pallida] | 2.2 |
| 75 | Translationally-controlled tumor protein-like [Exaiptasia pallida] | 1.21 |
| 76 | 14-3-3-like protein 2 [Exaiptasia pallida] | 1.49 |
| 77 | 14-3-3 protein zeta [Exaiptasia pallida] | 1.33 |
| 80 | Rho GDP-dissociation inhibitor 1 [Exaiptasia pallida] | 1.51 |
| 90 | GTPase KRas [Exaiptasia pallida] | 1.78 |
| 94 | Major antigen [Exaiptasia pallida] | 1.61 |
| 97 | Programmed cell death protein 6 [Exaiptasia pallida] | 3.55 |
| 106 | Myosin regulatory light chain 12B [Exaiptasia pallida] | 3.14 |
| 140 | E3 ubiquitin-protein ligase DZIP3 [Exaiptasia pallida] | 1.77 |

**Proteins downregulated in anemones colonized by *D. trenchii* during the day represented in Figure 5D**

| Day | | |
| --- | --- | --- |
| Gel ID | Protein Identity | Expression Ratio |
| 1 | Myosin heavy chain, striated muscle [Exaiptasia pallida] | -2.06 |
| 2 | Myosin heavy chain, striated muscle [Exaiptasia pallida] | -1.39 |
| 3 | Endoplasmin [Exaiptasia pallida] | -1.18 |
| 4 | Spectrin alpha chain, non-erythrocytic 1 [Exaiptasia pallida] | -1.46 |
| 8 | hypothetical protein AC249_AIPGENE16477 [Exaiptasia pallida] | -1.9 |
| 12 | Calpain-B [Exaiptasia pallida] | -1.48 |
| 13 | Phosphoenolpyruvate carboxykinase [GTP], mitochondrial [Exaiptasia pallida] | -1.65 |
| 18 | Protein disulfide-isomerase [Exaiptasia pallida] | -1.23 |
| 19 | Neuronal pentraxin-2 [Exaiptasia pallida] | -1.96 |
| 37 | Calpain-B [Exaiptasia pallida] | -1.48 |
| 38 | Alpha-enolase [Exaiptasia pallida] | -1.35 |
| 44 | Fumarylacetoacetase [Exaiptasia pallida] | -1.49 |
| 45 | Heat shock cognate 71 kDa protein [Exaiptasia pallida] | -1.66 |
| 59 | Malate dehydrogenase, cytoplasmic [Exaiptasia pallida] | -1.82 |
| 69 | Inactive pancreatic lipase-related protein 1 [Exaiptasia pallida] | -1.87 |
| 78 | Peptidyl-prolyl cis-trans isomerase FKBP14 [Exaiptasia pallida] | -1.14 |
| 79 | 14-3-3 protein zeta [Exaiptasia pallida] | -1.45 |
| 84 | S-crystallin 4 [Exaiptasia pallida] | -1.26 |
| 92 | Golgi-associated plant pathogenesis-related protein 1 [Exaiptasia pallida] | -2.69 |
| 93 | Sorcin [Exaiptasia pallida] | -3.09 |
| 94 | Major antigen [Exaiptasia pallida] | -1.48 |
| 110 | Actin, cytoplasmic [Exaiptasia pallida] | -2.31 |
| 112 | ADP-ribosylation factor 1 [Exaiptasia pallida] | -1.56 |
| 119 | hypothetical protein AC249_AIPGENE3884 [Exaiptasia pallida] | -1.3 |
| 121 | Ectin [Exaiptasia pallida] | -1.83 |
| 122 | hypothetical protein AC249_AIPGENE3884 [Exaiptasia pallida] | -1.15 |
| 125 | Myophilin [Exaiptasia pallida] | -1.4 |
| 132 | hypothetical protein AC249_AIPGENE7839 [Exaiptasia pallida] | -1.68 |
| 136 | Myotrophin [Exaiptasia pallida] | -1.63 |

**Proteins downregulated in anemones colonized by *D. trenchii* during the night represented in Figure 5D**

| Night | | |
| --- | --- | --- |
| Gel ID | Protein Identity | Expression Ratio |
| 1 | Myosin heavy chain, striated muscle [Exaiptasia pallida] | -1.17 |
| 8 | hypothetical protein AC249_AIPGENE16477 [Exaiptasia pallida] | -1.47 |
| 13 | Phosphoenolpyruvate carboxykinase [GTP], mitochondrial [Exaiptasia pallida] | -1.66 |
| 16 | hypothetical protein AC249_AIPGENE16868 [Exaiptasia pallida] | -1.29 |
| 19 | Neuronal pentraxin-2 [Exaiptasia pallida] | -1.86 |
| 21 | Lysostaphin [Exaiptasia pallida] | -1.44 |
| 26 | Tubulin alpha-1C chain [Exaiptasia pallida] | -1.35 |
| 31 | Selenium-binding protein 1 [Exaiptasia pallida] | -1.35 |
| 36 | hypothetical protein AC249_AIPGENE13469 [Exaiptasia pallida] | -1.21 |
| 37 | Calpain-B [Exaiptasia pallida] | -1.77 |
| 38 | Alpha-enolase [Exaiptasia pallida] | -1.54 |
| 44 | Fumarylacetoacetase [Exaiptasia pallida] | -1.58 |
| 45 | Heat shock cognate 71 kDa protein [Exaiptasia pallida] | -1.42 |
| 50 | Actin, cytoplasmic [Exaiptasia pallida] | -1.23 |
| 55 | Fructose-bisphosphate aldolase C [Exaiptasia pallida] | -1.53 |
| 56 | Serine--pyruvate aminotransferase, mitochondrial [Exaiptasia pallida] | -1.38 |
| 59 | Malate dehydrogenase, cytoplasmic [Exaiptasia pallida] | -1.42 |
| 69 | Inactive pancreatic lipase-related protein 1 [Exaiptasia pallida] | -1.73 |
| 79 | 14-3-3 protein zeta [Exaiptasia pallida] | -1.5 |
| 82 | Actin, cytoplasmic [Exaiptasia pallida] | -1.25 |
| 92 | Golgi-associated plant pathogenesis-related protein 1 [Exaiptasia pallida] | -2.3 |
| 93 | Sorcin [Exaiptasia pallida] | -3.03 |
| 110 | Actin, cytoplasmic [Exaiptasia pallida] | -2.3 |
| 112 | ADP-ribosylation factor 1 [Exaiptasia pallida] | -1.32 |
| 119 | hypothetical protein AC249_AIPGENE3884 [Exaiptasia pallida] | -1.54 |
| 121 | Ectin [Exaiptasia pallida] | -1.81 |
| 122 | hypothetical protein AC249_AIPGENE3884 [Exaiptasia pallida] | -1.29 |
| 128 | Rho GDP-dissociation inhibitor 1 [Exaiptasia pallida] | -1.15 |
